# The Excited-State Lifetime of Poly(NDI2OD-T2) Is Intrinsically Short

**DOI:** 10.1021/acs.jpcc.4c00653

**Published:** 2024-04-03

**Authors:** Melissa K. Gish, Chamikara D. Karunasena, Joshua M. Carr, William P. Kopcha, Ann L. Greenaway, Aiswarya Abhisek Mohapatra, Junxiang Zhang, Aniruddha Basu, Victor Brosius, Saied Md Pratik, Jean-Luc Bredas, Veaceslav Coropceanu, Stephen Barlow, Seth R. Marder, Andrew J. Ferguson, Obadiah G. Reid

**Affiliations:** † Materials, Chemical, and Computational Science Directorate, National Renewable Energy Laboratory, Golden, Colorado 80401, United States; ‡ Department of Chemistry and Biochemistry, The University of Arizona, Tucson, Arizona 85721-0041, United States; § Renewable and Sustainable Energy Institute, University of Colorado Boulder, Boulder, Colorado 80309, United States; ∥ Department of Chemical and Biological Engineering, University of Colorado Boulder, Boulder, Colorado 80309, United States; ⊥ Department of Chemistry, University of Colorado Boulder, Boulder, Colorado 80309, United States

## Abstract

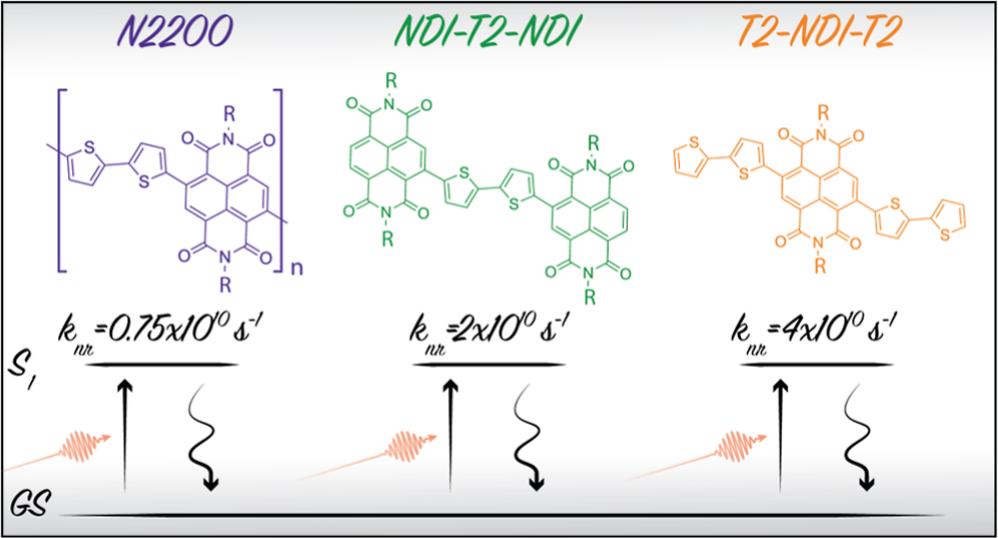

Conjugated polymers composed of alternating electron donor and acceptor segments have come to dominate the materials being considered for organic photoelectrodes and solar cells, in large part because of their favorable near-infrared absorption. The prototypical electron-transporting push–pull polymer poly(NDI2OD-T2) (N2200) is one such material. While reasonably efficient organic solar cells can be fabricated with N2200 as the acceptor, it generally fails to contribute as much photocurrent from its absorption bands as the donor with which it is paired. Moreover, transient absorption studies have shown N2200 to have a consistently short excited-state lifetime (∼100 ps) that is dominated by a ground-state recovery. In this paper, we investigate whether these characteristics are intrinsic to the backbone structure of this polymer or if these are extrinsic effects from ubiquitous solution-phase and thin-film aggregates. We compare the solution-phase photophysics of N2200 with those of a pair of model compounds composed of alternating bithiophene (T2) donor and naphthalene diimide (NDI) acceptor units, NDI-T2-NDI and T2-NDI-T2, in a dilute solution. We find that the model compounds have even faster ground-state recovery dynamics (τ = 45, 27 ps) than the polymer (τ = 133 ps), despite remaining molecularly isolated in solution. In these molecules, as in the case of the N2200 polymer, the lowest excited state has a T2 to NDI charge-transfer (CT) character. Electronic-structure calculations indicate that the short lifetime of this state is due to fast nonradiative decay to the ground state (GS) promoted by strong CT–GS electronic coupling and strong electron-vibrational coupling with high-frequency (quantum) normal modes.

## Introduction

So-called push–pull polymers^1−4^ drove the first wave of improvements in organic solar cell performance after efficiency gains from homopolymer–fullerene blends stagnated in the late 2000s.^5−9^ As such, these materials are likely to form the basis of future photoelectrochemical cells as well,^10−12^ particularly in polymer/polymer blends where both the electron-donating and electron-accepting polymers share this structural motif. N2200 has emerged as the prototypical electron-transporting push–pull conjugated polymer; it comprises a monomer composed of alternating naphthalene diimide (NDI) and bithiophene (T2) moieties and is employed extensively in both all-polymer organic solar cells^13,14^ and organic electrochemical and field-effect transistors.^15,16^

Although N2200 has good stability,^17^ charge transport properties,^15^ and redox potentials^18^ for these applications, its use as a solar absorber material in photovoltaics has been hampered by a short excited-state lifetime^19−21^ and an observable deficiency in quantum efficiency in spectral regions where N2200 absorbs exclusively.^13,22,23^ While low photoluminescence quantum yield^20^ and shortened excited-state lifetimes are typical of molecular and polymer aggregates, the case demonstrated in N2200 is extreme as there is complete ground-state (GS) recovery within 150 ps, both in thin-film and solution-phase experiments. The latter is especially surprising^20^ given that similar donor-NDI dyads and triads have been shown to have either significant fluorescence quantum yields or large triplet yields depending on the structure.^24^

In this paper, we show that these properties are intrinsic to the NDI-T2 repeat unit and not connected with ubiquitous solution phase aggregation phenomena, though these are certainly also present.^13,15,18^ Using a pair of model compounds that are well dispersed as isolated molecules in solution, having donor–acceptor–donor (DAD, T2-NDI-T2) and acceptor–donor–acceptor (ADA, NDI-T2-NDI) structures, we show via transient absorption (TA), time-resolved photoluminescence, spectroelectrochemistry, and electronic-structure calculations that the lowest-lying intramolecular charge-transfer (CT) state relaxes very quickly nonradiatively back to the GS as a result of strong CT–GS electronic coupling and strong electron-vibrational coupling with high-frequency vibrational modes.

## Methods

### Sample Preparation

Samples for TA were prepared in a nitrogen glovebox and sealed in a 2 mm cuvette.

### Transient Absorption

TA experiments were completed using a Ti/sapphire regenerative amplifier with an 800 nm fundamental and 90 fs pulse width (Coherent Astrella). The output was split with a majority of the power going to an optical parametric amplifier (Light Conversion, TOPAS-C) to generate our pump pulse (525 or 700 nm). The smaller portion of the beam traveled through a mechanical delay stage and focused into a sapphire crystal to generate our white light probe pulse via supercontinuum generation. The white light probe used in this study spans from 440 nm through 850 nm. The probe is delayed relative to the pump pulse using the mechanical delay stage. Pump and probe are focused and spatially overlapped at the sample. A portion of the generated white light is redirected prior to reaching the sample to a reference detector to reduce noise to <0.1 mOD. A fiber coupled multichanneled spectrometer with a CMOS sensor was used to monitor changes in the probe spectrum. Ultrafast Systems-provided software, Helios and Surface Xplorer, were used to collect and analyze the data, respectively. Data were chirp-corrected.

### Time-Resolved Photoluminescence

Optical excitation with ca. ∼20 ps pulses at 550 nm for the model compounds and 700 nm for N2200 was supplied by an NKT supercontinuum fiber laser (SuperK EXU-6-PP) with a 2.89 MHz repetition rate. A 10 nm bandpass filter was used to reduce the spectral bandwidth of the excitation beam. A Hamamatsu 300–900 nm (C10910-04) streak camera was used to collect time-resolved PL spectra. Instrument response was captured by scattering some excitation light into the detector using ground glass in the sample position. Transients were analyzed at the wavelength of maximum PL intensity for each film.

### Spectroelectrochemistry

All (spectro)electrochemical measurements were performed in an inert glovebox environment (<1 ppm of O_2_) equipped with electrical and fiber couple feedthroughs, driven by a BioLogic SP-150e potentiostat and recorded using BioLogic software. All presented (spectro)electrochemical data follow the International Union of Pure and Applied Chemistry convention. For spectroelectrochemistry, optical absorption spectra were simultaneously collected by Ocean Insight software, using an Ocean Optics halogen broadband white light source and an Ocean Insight Flame Series miniature UV–vis spectrometer. Typical exposure times were 10–20 ms with 50–100 averages to prevent detector saturation.

For both T2-NDI-T2 and NDI-T2-NDI, analyte concentrations were ∼1 mM in 4:1 dichlorobenzene/acetonitrile with 0.1 M NBu_4_^+^PF_6_^–^ (Sigma-Aldrich >99% electrochemical grade) as a supporting electrolyte. Spectroelectrochemical measurements were performed in a thin-layer quartz cuvette using a ceramic honeycomb electrode designed to increase the effective optical path length (Pine Research #AKSTCKIT3). The working and counter electrodes were Pt on a printed ceramic support and a separate nitric acid-treated Ag wire was used as a pseudoreference. To collect the spectra of the reduced compounds, the working electrode was held at a potential more reducing (negative) than the targeted reduction potential until the optical absorption spectra had stabilized, at least 45 s. Absorption spectra were also collected simultaneously with cyclic voltammetry (CV), demonstrating the reversibility of the reduction processes (Supporting Information Figure S5.1).

Additional CVs were performed in a 2 mL electrochemical cell and ceramic printed electrode (with Pt working and counter electrodes, Pine Research #AKSPEKIT; same Ag pseudoreference and electrolyte compositions). CVs were scanned at 20 mV/s, starting at the open circuit potential, scanning negative to −1.75 V and then scanning positive to 0.5 V vs the Ag pseudoreference, and repeating three times. Subsequently, 40 μL each of 20 mM ferrocene (Fc) and ferrocenium (Fc^+^) tetrafluoroborate in acetonitrile solutions were added to the electrolyte and the CVs were repeated in order to reference the analyte reduction potentials to the Fc/Fc^+^*E*_1/2_.

### Theoretical Methods

The GS geometries and vibrational frequencies of isolated NDI-T2, NDI-T2-NDI, and T2-NDI-T2 molecules were studied at the LC-ωhPBE/6-31G(d,p) level of theory. The range-separation parameter (ω) was tuned following the common procedure detailed elsewhere.^25−27^ The tuned range separation parameter was set to 0.1553 Bohr^–1^ for all systems. Time-dependent DFT (TD-DFT) calculations within the Tamm–Dancoff approximation were carried out to compute the excited-state properties and estimate the electron-vibration and spin–orbit couplings. In order to account for the effect of the solvent (oDCB) on the electronic properties, we considered implicitly the solvent dielectric medium by employing the linear-response polarizable-continuum model.^28^ In addition, the CT absorption transition energies were corrected using a state-specific solvation procedure based on the corrected linear-response approach.^29^ All DFT calculations were performed with the Gaussian 16 software.^30^

The nonradiative decay rate constant from the CT state to GS was derived in the framework of the Marcus–Levich–Jortner (MLJ) approach^31^
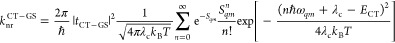
1Here, *t*_CT–GS_ and *E*_CT_ represent the electronic coupling between the CT state and ground state and the adiabatic CT energy, respectively; *S*_*qm*_ and ω_*qm*_ are the effective Huang–Rhys factor and vibrational frequency, respectively, which define the quantum contribution (λ_*qm*_ = *S*_*qm*_ω_*qm*_) to the reorganization energy due to high-frequency vibrational modes; λ_c_ denotes the classical part of the reorganization energy due to low-frequency modes. The *S*_*qm*_, ω_*qm*_, and λ_c_ parameters were derived in the framework of the MLJ approach by fitting the reduced emission band of NDI-T2-NDI measured in toluene. The generalized Mulliken–Hush model was used to estimate *t*_CT–GS_(32)
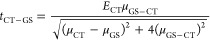
2Here, μ_CT_ and μ_GS_ are the dipole moments of the CT and GSs and μ_GS–CT_ is the transition dipole moment between these two states. In the estimation of *t*_GS–CT_, we used the DFT results obtained for NDI-T2. The derived coupling is about 0.5 eV. In addition, the nonradiative decay rate constant from the CT state to GS was estimated in the framework of the nonadiabatic coupling (NAC) model^33,34^ and using the thermal vibration correlation function^35−37^ formalism as implemented in the MOMAP package.^38^

## Results and Discussion

**Figure 1:**
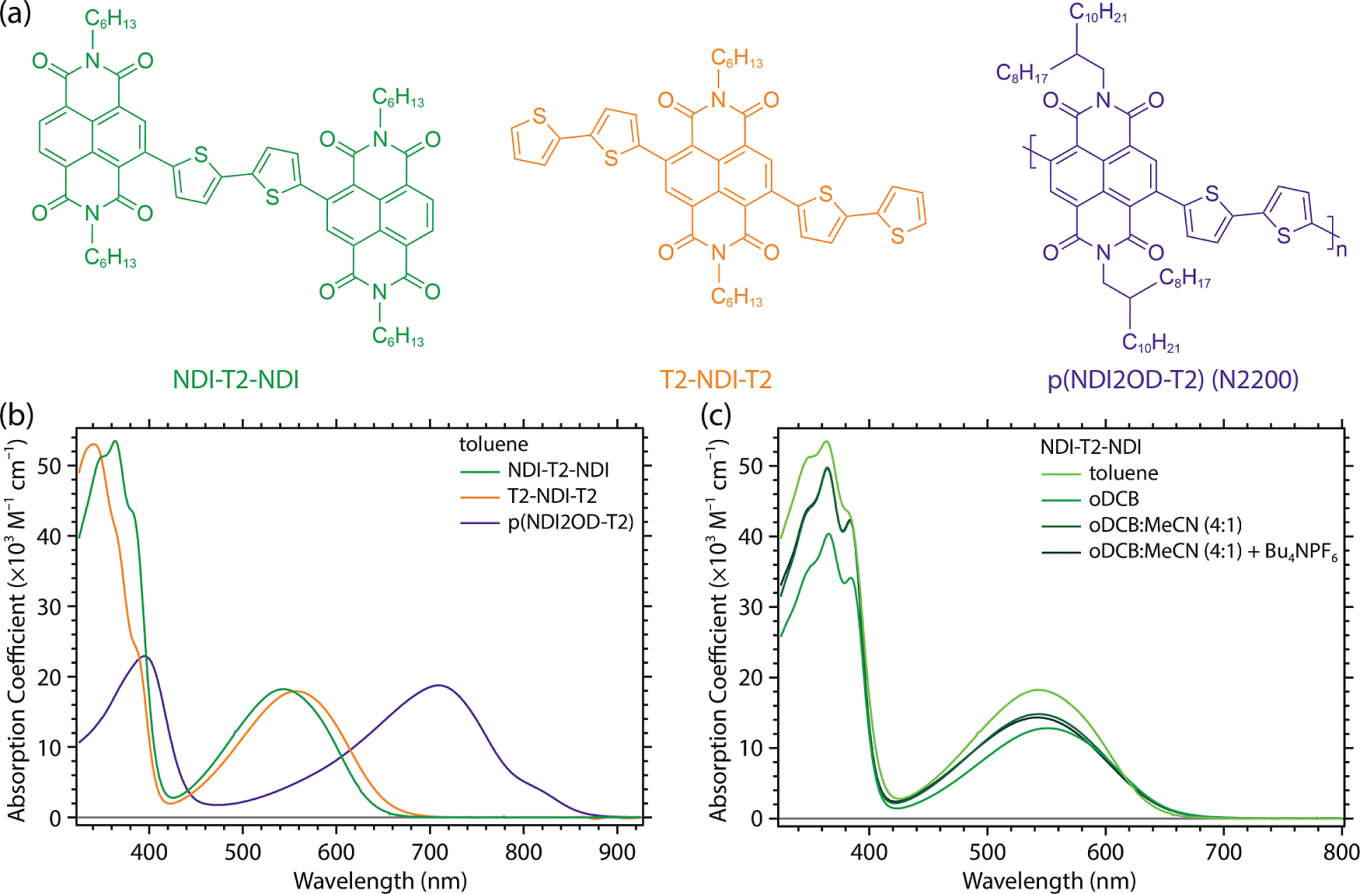
(a) Chemical structures and (b) absorption spectra of the NDI-T2 model compounds and the prototypical NDI2OD-T2 polymer, N2200. (c) Absorption spectra of the NDI-T2-NDI model compound in toluene, ortho-dichlorobenzene (oDCB), and a mixed solvent system comprising ortho-dichlorobenzene and acetonitrile (oDCB/MeCN) either with or without tetrabutylammonium hexafluorophosphate (Bu_4_N^+^PF_6_^–^) salt.

Figure 1a shows the molecular structures of the compounds examined in this study: 2,2′-(2,2-bithiophene-5,5′-diyl)bis(*N,N*′-bis(*n*-hexyl)naphthalene-1,8:4,5-bis(dicarboximide)) (NDI-2T-NDI), *N,N*′-di-*n*-hexyl-2,6-di(2,2′-bithiophen-5-yl)naphthalene-1,8:4,5-bis(dicarboximide)(2T-NDI-2T), and poly(*N,N′*-di(2-octyldodecyl)naphthalene-1,8:4,5-bis(dicarboximide)-2,6-diyl)-*alt*-(2,2-bi-thiophene-5,5′-diyl) (N2200). The T2-NDI-T2 and NDI-T2-NDI model compounds were synthesized according to a procedure described previously,^18^ while the N2200 polymer was obtained commercially from 1-Material. The calculated molar absorptivities for T2-NDI-T2, NDI-T2-NDI, and N2200 dissolved in toluene are listed in Figure 1b. Note that the absorption coefficient for N2200 is calculated using the molecular weight of the NDI-T2 monomer. Representative absorption spectra of NDI-T2-NDI in various solvent systems, including a mixed ortho-dichlorobenzene:acetonitrile (oDCB/MeCN) solvent with and without tetrabutylammonium hexafluorophosphate (Bu_4_N^+^PF_6_^–^), are shown in Figure 1c.

The absorption spectra of the model compounds are qualitatively similar to the spectrum for N2200 in solution, except for a marked hypsochromic shift of the dominant absorption bands. The lowest energy absorption band, centered at ca. 550 nm for the molecular model compounds and at ca. 710 nm for N2200, is attributed to a CT transition that moves the electron density from the T2 moiety to the NDI chromophore during photoexcitation. This assignment is fully supported by TD-DFT calculations performed on NDI-T2, NDI-T2-NDI, and T2-NDI-T2 compounds (see Tables S7.1–S7.3 in the Supporting Information). The natural transition orbitals (Figure S7.1) derived for the S_0_ → S_1_ transition clearly show that the lowest excited state in all three oligomers has a dominant CT character. We note that the CT state in NDI-T2-NDI is characterized by two electronic configurations, i.e., NDI^–^-T2^+^-NDI and NDI-T2^+^-NDI^–^. The CT state formed immediately upon excitation (at the GS geometry) can therefore be described as a symmetrical linear combination of these two configurations. The same applies for the CT state in T2-NDI-T2, which possesses both T2^+^-NDI^–^-T2 and T2-NDI^–^-T2^+^ electronic configurations. The DFT calculations indicate that the electronic coupling between these two localized electronic configurations is about 0.1 eV, while the related relaxation energies are about 0.4 eV. As a result, after formation of the CT state, the system is expected to undergo a fast symmetry-breaking transition into one of the two geometrically relaxed electronic configurations mentioned above. The higher energy absorption bands are attributed to a superposition of electronic π – π* transitions of the T2 and NDI moieties. These are higher in absorptivity relative to N2200 since the NDI-T2-NDI and T2-NDI-T2 model compounds consist of ca. 1.5 N2200 monomer units. Previous experimental studies on N2200 and related oligomers have suggested that the rise in relative CT band intensity and its red-shifting result from the combination of (i) increased along-chain delocalization and (ii) increased chain aggregation with increased chain length.^18,39,40^

In thin films, the spectrum of N2200 is barely altered relative to the solution phase, suggesting strong chain–chain interactions in solution. In contrast, thin films of NDI-T2-NDI and T2-NDI-T2 both dramatically red shift relative to their solution-phase spectra, coming to more closely resemble the spectrum of N2200 (see Figure S4.1). These observations lend further credence to the assertion that the NDI-T2-NDI and T2-NDI-T2 model compounds are well-solvated in solution and that the red-shifted CT bands observed in thin films indicate the presence of interchromophore CT transitions (or interchain transitions in the case of N2200) promoted by molecular aggregation.

**Figure 2:**
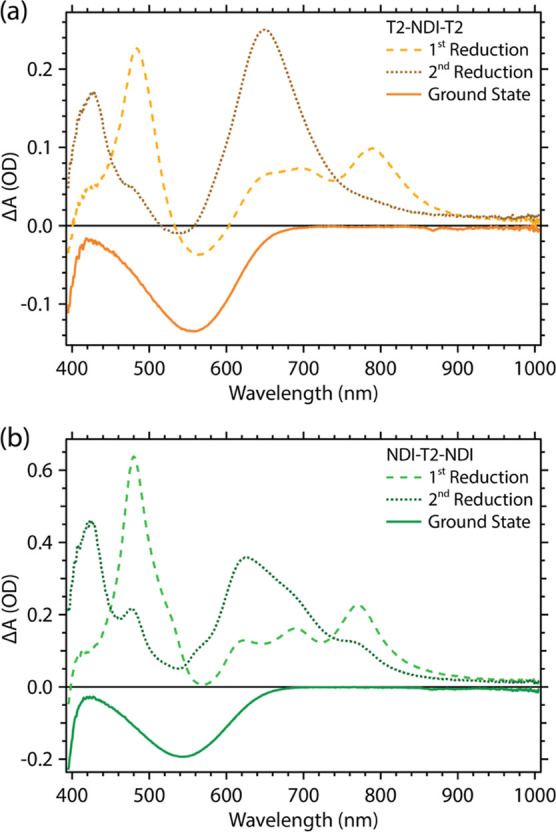
Difference absorbance spectra corresponding to the first and second reductions of the (a) T2-NDI-T2 and (b) NDI-T2-NDI model compounds, collected in a 4:1 oDCB/MeCN electrolyte after stabilization of the spectra under applied potential. The GS absorbance spectra are inverted to illustrate the position of the expected GS absorbance bleach in the spectroelectrochemical data. T2-NDI-T2 spectra collected at −1.30 and −1.70 V vs Fc/Fc^+^ for first and second reductions, respectively. NDI-T2-NDI spectra collected at −1.47 and −1.82 V vs Fc/Fc^+^ for first and second reductions.

Both T2-NDI-T2 and NDI-T2-NDI undergo two reversible reductions, with half-wave potentials, *E*_1/2_, of −0.98 and −1.49 V vs ferrocene/ferrocenium (Fc/Fc^+^) for T2-NDI-T2 and −1.08 and −1.53 V vs Fc/Fc^+^ for NDI-T2-NDI (CV in Figure S5.1; irreversible oxidations at positive potentials are not shown). Figure 2a,b shows UV–vis absorption spectra collected simultaneously with chronoamperometry at potentials slightly negative of the first and second reductions for each compound, along with their respective inverted GS absorption spectrum. These data provide a useful point of comparison with the TA studies described below, given the CT character of the lowest energy excited state. The first reduction spectra of the two compounds are broadly similar, with prominent reduced-state absorption bands over 600–800 and 400–500 nm as well as relatively weak bleach bands near 550 nm; the second reduction spectra of both show increased absorption near 450 nm and a blue shift of the broad absorption over 600–800 nm as well as the bleach bands. It is worth noting that the reduced spectra are similar to, but considerably broader than, those of simple NDI^•–^ and NDI^2–^ species, presumably due to electronic and/or steric effects of the T2 groups on the NDI cores.^41^ The similarity between the first and second reduction spectra of NDI-T2-NDI with those of T2-NDI-T2 suggests that the NDI-T2-NDI accepts two electrons at each reduction, such that each NDI unit is reduced to NDI^•–^ (or twice-reduced to NDI^2–^) rather than a delocalization of the electrons over the whole molecule, which would afford very different spectra than the T2-NDI-T2; such decoupled behavior of connected NDI units has previously been observed in similar compounds.^42,43^

**Figure 3:**
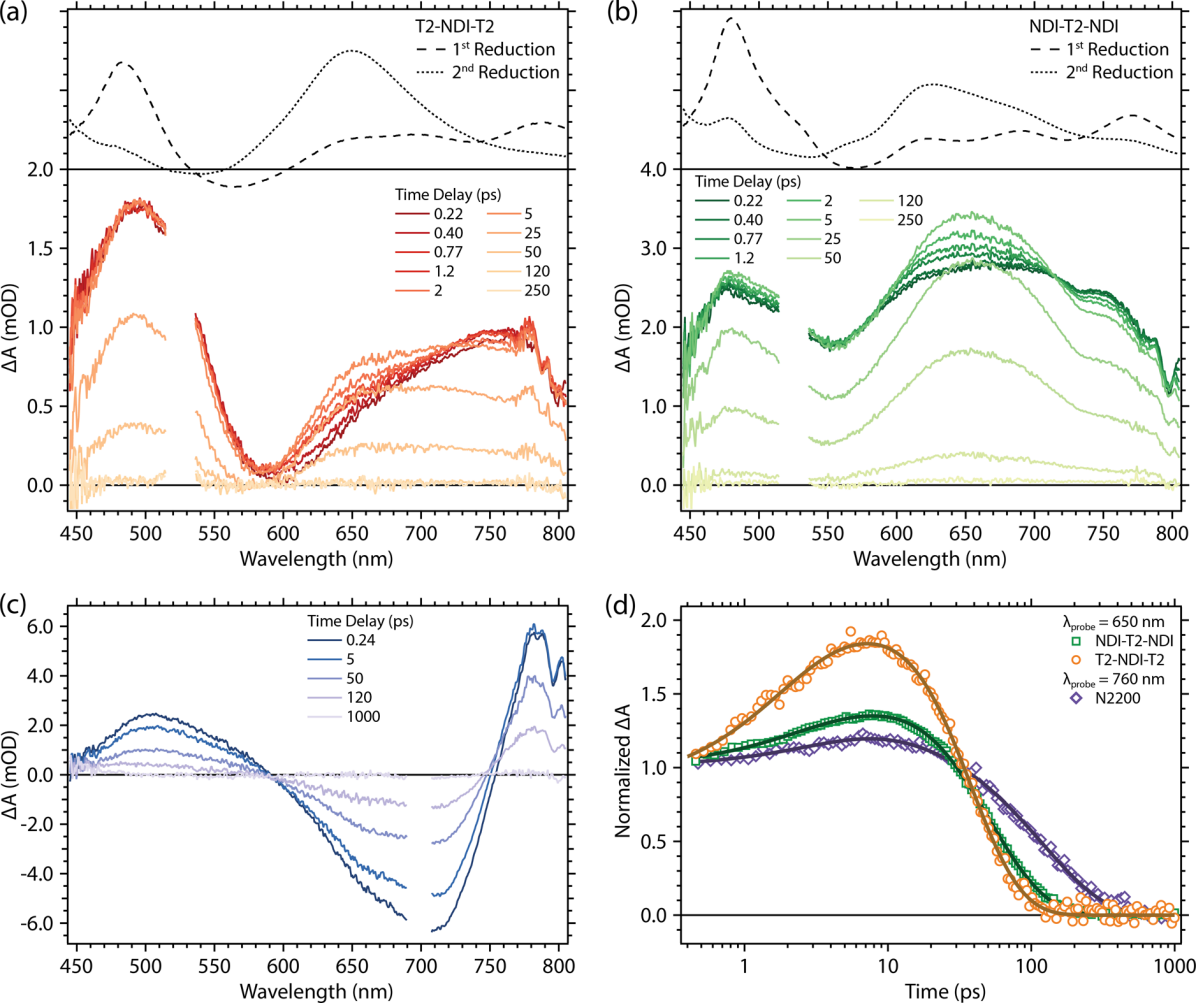
TA spectra in oDCB solution of (a) T2-NDI-T2, (b) NDI-T2-NDI, and (c) N2200. (a) and (b) were photoexcited at 525 nm (180 nJ/pulse) while (c) was photoexcited at 700 nm (100 nJ/pulse). The spectra of the reduced model compounds as determined via spectroelectrochemistry in a 4:1 oDCB/MeCN mixture are shown as a black dashed line in (a) and (b). (d) Comparison of the excited-state kinetics probed at the 650 nm band for NDI-T2-NDI, T2-NDI-T2, and 760 nm band for N2200. Fits are shown as black lines.

TA spectra of T2-NDI-T2 and NDI-T2-NDI photoexcited into the intramolecular CT band at 525 nm are shown in Figure 3a,b respectively, and a summary of kinetic fit parameters is given in Table 1 as well as in Tables S6.1 and S6.2. Both model compounds contain a broad photoinduced absorption (PIA) across the observed spectral window. Similar to the absorption spectra collected under the applied potential in Figure 2, the ground-state bleach (GSB) of the intramolecular CT absorption does not appear to be negative because of the aforementioned broad PIA superimposed on top of it. Within the first few ps after photoexcitation, there is a rapid evolution of the spectrum for both compounds, resulting in a PIA growth evident between 600 and 700 nm. The spectra of the first reduction and second reduction species for T2-NDI-T2 and NDI-T2-NDI, measured via spectroelectrochemistry, are superimposed for comparison as black dashed and dotted lines, respectively.

**Table 1:** Multi-Exponential Fit Parameters from the Fits in Figure 3d

	N2200, oDCB (λ_probe_ = 760 nm)	NDI-T2-NDI, oDCB (λ_probe_ = 650 nm)	T2-NDI-T2, oDCB (λ_probe_ = 650 nm)
*A*_1_ (rise)	0.18	0.08	0.13
τ_1_, ps (rise)	4.8 (±0.4)	1.95 (±0.3)	1.62 (±0.3)
*A*_2_ (rise)		0.25	0.30
τ_2_, ps (rise)		9.8 (±0.8)	9.7 (±1.6)
*A*_3_ (decay)	0.82	0.67	0.57
τ_3_, ps (decay)	133 (±1.8)	45 (±0.6)	27 (±1.3)

The PIA growth created upon 525 nm photoexcitation in both cases closely resembles the electrochemically generated spectra corresponding to the first reductions. Since the lowest excited state of each compound is expected to be an intramolecular CT state, formally consisting of T2^+^-NDI^–^, it is reasonable to expect that the excited-state spectra should bear some resemblance to the electrochemically reduced species: T2-NDI^•–^. There is a secondary evolution exhibited by both compounds within 5–10 ps, where the PIA centered at 650 nm grows concomitantly with a decay at 760 nm. We note that some initial evolution may occur within our instrument response, and we normalized the data immediately after the instrument response based on the parameters of the excitation pulse. Figures S6.3 and S6.4 show the kinetics on a linear scale with a simulated instrument response. The data are normalized and the fits begin at ca. 350–400 fs after this instantaneous rise. After this evolution is complete, the spectra resemble a linear combination of the first and and second reduction spectra. This is surprising since TA is a single-photon excitation experiment, and although dianion formation has been observed in thin films,^44^ the evolution here is too fast for a second electron transfer to occur *inter*molecularly via diffusion-controlled collisions. There are three possibilities that could explain this observation. First, it could be coincidental. Due to the oxidative instability of these compounds, we cannot evaluate what contribution the T2^+^ fragment makes to the spectrum. Second, an excited-state structural relaxation after the initial excitation may be precipitated by the symmetry breaking that spontaneously localizes the excited-state from an initial linear combination of the two possible CT configurations already discussed to a single localized configuration such as T2-NDI^–^-T2^+^. Conformational similarity between T2-NDI^–^-T2^+^ and T2-NDI^2–^-T2 could explain the resemblance between their spectra. Third, it is possible that these picosecond spectral dynamics are connected with an equilibration between the ^3^CT and ^1^CT manifolds, as has been invoked previously to explain fast intersystem crossing in similar chromophores.^24^ Thus, far, our data are not sufficient to definitively distinguish these possibilities. However, it is notable that we do not detect significant formation of long-lived local triplet states here, making the intersystem crossing hypothesis less likely.

The TA spectra for the N2200 photoexcited at the CT band at 700 nm are shown in Figure 3c for comparison with the model compounds discussed above. The N2200 excited-state spectrum consists of a strong GSB bordered by two PIA features at 500 and 760 nm. Similar to the PIA at 600 nm for the model compounds, the PIA for N2200 at 760 nm grows in intensity over the first few ps. This is clearly evident when normalizing the TA spectra to the GSB at 700 nm and in the kinetics at a probe wavelength of 760 nm (Figure S6.1), showing that the behavior of N2200 is qualitatively similar to that of the model compounds.

The kinetics associated with the PIA bands of NDI-T2-NDI and T2-NDI-T2 at a probe wavelength of 650 nm and the N2200 PIA probed at 760 nm are shown in Figure 3d. The average rise time associated with the growth of the PIA is equal for both model compounds (τ_avg_ = 6 ps) and is slightly faster in the case of N2200 (τ = 4.8 ps). The magnitude of the growth of the PIA is larger for T2-NDI-T2 than for either NDI-T2-NDI or N2200, suggesting a larger excited-state evolution for T2-NDI-T2. In all cases, the excited-state lifetimes are short (27 ps for T2-NDI-T2, 45 ps for NDI-T2-NDI, and 133 ps for N2200). The shorter excited-state lifetime of T2-NDI-T2 could be due to greater flexability of the T2-NDI-T2 molecule that acclerates the torsional/vibrational deactivation of the excited state. The excited-state lifetime is ca. 3–5 times longer for the polymer compared to the model compounds, possibly due to inhibited torsional relaxation in N2200 as well as the possibility of CT between aggregates formed by interacting chain segments.

**Figure 4:**
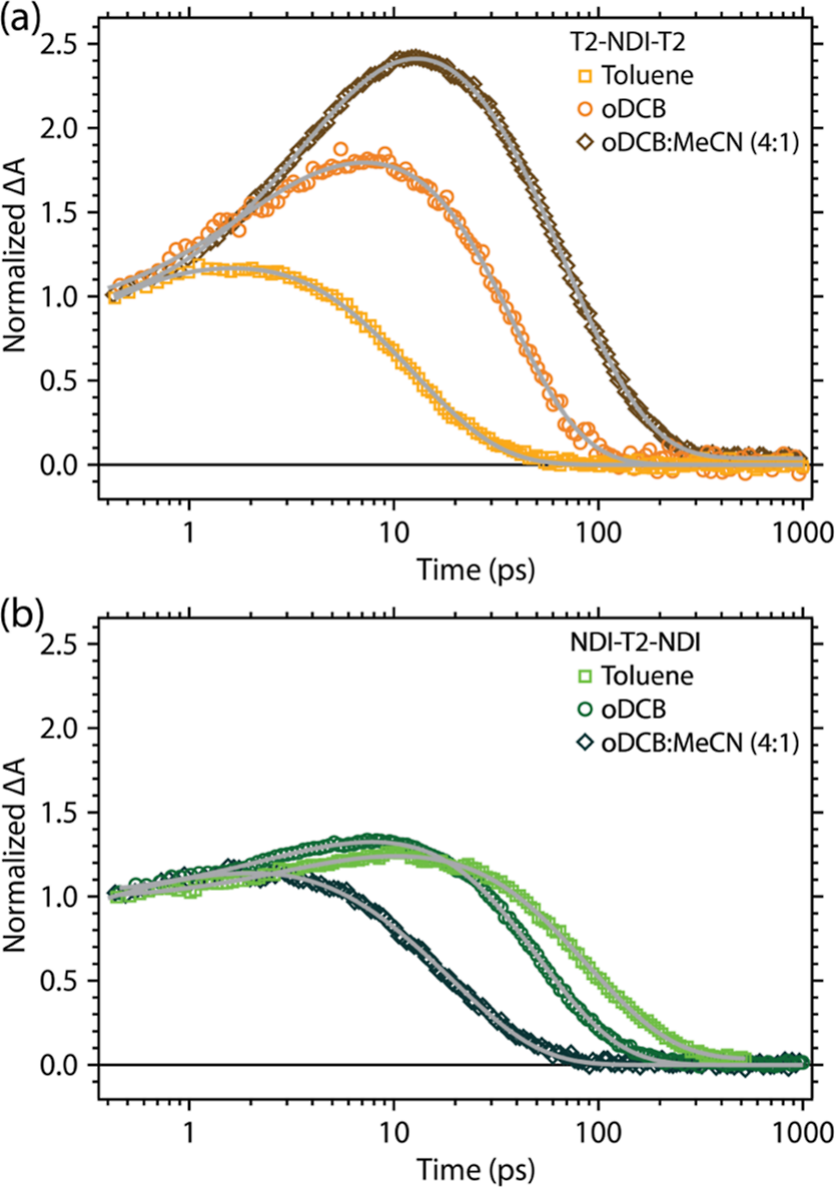
Normalized TA kinetics of (a) T2-NDI-T2 and (b) NDI-T2-NDI probed at 650 nm after 525 nm photoexcitation in oDCB (orange/green squares), 4:1 mixture of oDCB/MeCN (brown/dark green diamonds), and toluene (peach/lime green circles). Fits are shown as gray lines.

In order to understand the effect of the dielectric environment on the efficacy of charge separation and excited-state lifetime, we varied the solvent system used to dissolve the model (Figure 4), using toluene (ϵ_r_ = 2.4), oDCB (ϵ_r_ = 9.9) and a 4:1 mixture of oDCB and MeCN (ϵ_r_ ≈ 37). For T2-NDI-T2, in the 4:1 mixture of oDCB and MeCN, there is a significant increase in the magnitude of the growth at 650 nm (Figure 4a) and an extension of the excited-state lifetime to 64 ps, which is double the lifetime in oDCB alone. In toluene (with the lowest dielectric constant), this growth is inhibited and the lifetime decreases relative to that in oDCB. This behavior follows the trend in the dielectric constant and is consistent with our assignment that the growth of the PIA at 650 nm is due to localization of positive/negative charges on their respective donor/acceptor moieties due to the stabilizing action of the solvent. There is a stimulated emission contribution in toluene, most evident in the NIR TA spectra (Figure S6.2), that is not as significant in the oDCB/MeCN mixture, though still present to some degree.

NDI-T2-NDI shows markedly different behavior based on the solvent (Figure 4b). There are no notable changes in the growth amplitude probed at 650 nm based on the dielectric constant, and the longest excited-state lifetime is measured in toluene at 85 ps. We propose two competing pathways to reconcile the opposite trends observed for NDI-T2-NDI and T2-NDI-T2 as a function of solvent polarity: the relative influence of the reorganization energy for recombination and a decreasing driving force for recombination in the Marcus inverted regime. The pronounced spectral evolution and extended lifetime of T2-NDI-T2 are evidence of a significantly twisted intramolecular charge transfer state that is absent, or at least weaker, in the NDI-T2-NDI molecule. Increasing the solvent polarity stabilizes this species, and the increased reorganization energy for recombination of the twisted state slows the overall rate of excited-state decay. In contrast, the less pronounced excited-state reorganization of NDI-T2-NDI, combined with the dielectric stabilization of the CT state, serves to accelerate recombination due to a decreasing driving force for return to the GS in the Marcus inverted regime.

Thus, far, our results show unequivocally that the excited-state lifetime of N2200 is not limited by aggregation phenomena nor is it controlled in a meaningful way by the strength of the excited-state conformational changes we infer from the transient spectra: even the T2-NDI-T2 excited state only lives 67 ps in 4:1 oDCB/MeCN. We now turn to an investigation of the origin of the rapid excited-state decay we observe through a combination of theory and experiments.

The photoluminescence spectra of all three compounds (Figures S3.1 and S3.4) in dilute oDCB solution are remarkably similar in shape but with the N2200 spectrum red-shifted by ca. 300 meV (150 nm) relative to the NDI-T2-NDI and T2-NDI-T2 compounds. The emission profile for the model compounds does not significantly change as a function of the solvent; however, in toluene, the N2200 emission spectrum has a prominent vibronic progression (Figure S3.1). The radiative rate constant (*k*_r_) from the CT state to the ground state was derived within the Strickler–Berg approach using the experimental absorption and emission spectra. As illustrated in the Supporting Information (Figures S3.2 and S3.3), both NDI-T2-NDI and T2-NDI-T2 display similar values of *k*_r_, about 4 × 10^7^ s^–1^ in both oDCB and toluene solutions.

The nonradiative decay rate constants (*k*_nr_) were first evaluated in the framework of the MLJ approach (see the Theoretical Methods section). The electronic coupling parameter *t*_CT–GS_ was estimated in the framework of the generalized Mulliken–Hush model, while the vibrational parameters were derived by fitting the reduced emission band of NDI-T2-NDI measured in toluene (see Figure S7.2). We obtain the following parameters: *S*_*qm*_ = 1.03, ω_*qm*_ = 0.19 eV and λ_c_ = 0.2 eV. Taking into account that the electronic coupling is estimated to be about 0.5 eV, the MLJ approach yields a value of 3 × 10^10^ s^–1^ for *k*_nr_ in toluene. However, such a high value of the electronic coupling raises questions of whether the use of the perturbative MLJ approach is justified. Therefore, we also evaluated *k*_nr_ in the framework of the NAC model. Since it turned out that the excited-state geometries of the NDI-T2-NDI and T2-NDI-T2 do not converge, these calculations were then performed on a simpler NDI-T2 molecule. The related vibrational and NAC couplings are given in Tables S7.4 and S7.5, respectively. In that case, we obtained a value of 8 × 10^11^ s^–1^ for *k*_nr_ when considering an implicit oDCB dielectric medium. Overall, both MLJ and NAC calculations point toward very fast (ps) nonradiative decay processes in N2200 and its oligomers. Finally, using the MLJ approach and DFT-derived vibrational and spin–orbit couplings (Tables S7.6 and S7.7), we evaluated the rate constant for the intersystem crossing (ISC) transition from the S_1_ state to the T_1_ state in NDI-T2. The ISC rate constant was estimated to be on the order of 1 × 10^9^ s^–1^. Thus, while ISC is significantly faster than the radiative decay transition, it remains about 1-to-2 orders of magnitude slower than the nonradiative decay. These calculations are consistent with the lack of any evidence for a long-lived triplet population in our data.

## Conclusions

We have shown that the excited-state relaxation of N2200 is intrinsically fast and not connected with ubiquitous solution-phase aggregation of the polymer. Comparison of the solution-phase photophysics between N2200 and a pair of small-molecule models consisting of T2-NDI-T2 and NDI-T2-NDI fragments shows that the latter exhibits even faster relaxation than the N2200 polymer. Theoretical calculations of the ground- and excited-state electronic structure provide evidence that this fast nonradiative relaxation is mediated by strong coupling to intramolecular vibrational modes. A Strickler–Berg analysis suggests a radiative lifetime of 25 ns for both model compounds, which is consistent with the very weak luminescence observed experimentally when their experimental excited-state lifetime is considered. We also observe a previously unreported evolution in both N2200 and model compound excited-state spectra taking place in the first 10 ps after excitation. Both this spectral evolution and the overall lifetime of the complexes are modulated by their dielectric environment. The NDI-T2-NDI molecule exhibits a positive correlation between its excited-state lifetime and the dielectric constant of the solvent with little change in its spectral evolution. The T2-NDI-T2 molecule exhibits the opposite trend, with the longest lifetime observed in the smallest dielectric constant solvent and a strong increase in the amplitude of spectral evolution. We suggest that these differences are connected with a strongly twisted intramolecular charge transfer state in T2-NDI-T2, which is much weaker (although present) in both NDI-T2-NDI and N2200. Overall, these results show that while N2200 remains one of the better polymer acceptors for energy conversion applications, development of stable electron-transporting polymers that possess superior excited-state characteristics is still needed to fully realize the potential of all-polymer photoelectrodes and solar cells.

## Supporting Information Available

Details of the synthesis of the NDI-T2-NDI and T2-NDI-T2 compounds as well supporting theoretical, spectroscopic, and electrochemical data (PDF)
