# Supplementary material for: The Excited-State Lifetime of Poly(NDI2OD-T2) Is Intrinsically Short
Source: J Phys Chem C Nanomater Interfaces. 2024 Apr 3;128(15):6392–400. doi: 10.1021/acs.jpcc.4c00653 (PMC11033933; doi:10.1021/acs.jpcc.4c00653)
Supplement: Supplementary file 1 — jp4c00653_si_001.pdf [file jp4c00653_si_001.pdf]

Supporting Information for: The Excited-State Lifetime of Poly(NDI2OD-T2) is Intrinsically Short

Melissa K. Gish[1,*c], Chamikara D. Karunasena[2], Joshua M. Carr[3], William P. Kopcha[1], Ann L. Greenaway[1], Aiswarya Abhisek Mohapatra[3], Junxiang Zhang[3], Aniruddha Basu[3], Victor Brosius[3], Saied Md Pratik[2], Jean-Luc Bredas[2], Veaceslav Coropceanu[2], Stephen Barlow[3,1], Seth R.Marder[1,3,4,5], Andrew J. Ferguson[1,*b], and Obadiah G. Reid[1,3,*a]

[1]Materials, Chemical, and Computational Science Directorate, National Renewable Energy Laboratory. Golden CO 80401, USA
[2]Department of Chemistry and Biochemistry, The University of Arizona. Tucson AZ 85721-0041, USA
[3]Renewable and Sustainable Energy Institute, University of Colorado Boulder. Boulder CO 80309, USA
[4]Department of Chemistry, University of Colorado Boulder. Boulder CO 80309, USA
[5]Department of Chemical and Biological Engineering, University of Colorado Boulder. Boulder CO 80309, USA
[*a]obadiah.reid@colorado.edu
[*b]andrew.ferguson@nrel.gov
[*c]melissa.gish@nrel.gov

Contents

1 Synthesis of T2-NDI-T2 and NDI-T2-NDI: 2

1.1 T2-NDI-T2: 2

1.2 NDI-T2-NDI: 2

2 UV-Vis Spectroscopy Details: 7

3 Steady-State and Time-Resolved Photoluminescence: 8

3.1 Steady-State Luminescence Measurement Details: 8

3.2 Strickler-Berg Analysis: 10

3.3 TRPL Data: 12

4 Thin-Film Absorbance: 13

5 Cyclic Voltammetry: 14

6 Supplemental Transient Absorption Data: 15

7 Supplemental Calculations: 18

1 Synthesis of T2-NDI-T2 and NDI-T2-NDI:

T2-NDI-T2 and NDI-T2-NDI were synthesized following a previous report of analogous compounds with different N,N'-substituents;[1] the precursors NDI-Br_2 and NDI-Br were synthesized as previously reported[1–3] and the synthetic scheme is shown below.

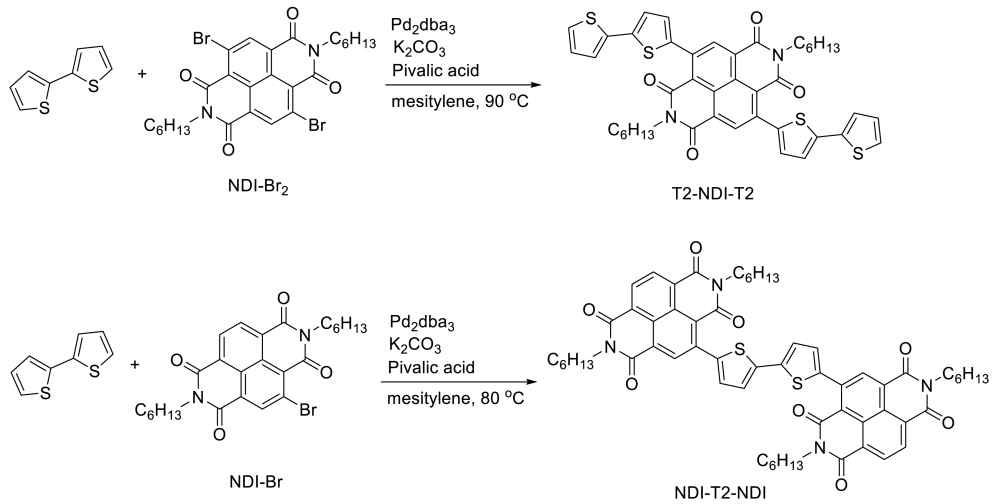

Figure S1.1: Synthetic scheme for T2-NDI-T2 and NDI-T2-NDI molecules.

1.1 T2-NDI-T2:

Bithiophene (280 mg, 1.69 mmol, 10 eq), NDI-Br_2 (100 mg, 169 μmol, 1.0 eq), K_2CO_3 (70.0 mg, 506 μmol, 3.0 eq), and pivalic acid (17.2 mg, 169 μmol, 1.0 eq) were weighed into a dry vial containing a stirring bar. Then 1.5 mL deoxygenated mesitylene was added under a nitrogen atmosphere and the reaction mixture was stirred for 5 min to fully dissolve the monomers. Then Pd_2dba_3 (15.5 mg, 16.7 μmol, 0.1 eq) was added under nitrogen at room temperature. The vial was sealed and placed into a preheated oil bath and stirred for 24 h at 90 °C. After cooling to room temperature, the reaction mixture was directly subjected to column chromatography to yield the desired product (46 mg, 36%). ^{1}H NMR (400 MHz, $CDCl_3$) δ 8.82 (s, 2H), 7.34–7.27 (m, 8H), 7.09 (dd, J = 5.1, 3.6 Hz, 2H), 4.16 (appt, J = 7.6 Hz, 4H), 1.76–1.67 (m, 4H), 1.37–1.30 (m, 12H), 0.90 (appt, J = 7.1 Hz, 6H). $^{13}C^1H$ NMR (101 MHz, $CDCl_3$) δ 162.14, 140.62, 139.51, 139.27, 136.78, 136.56, 129.86, 127.98, 127.54, 125.44, 125.22, 124.47, 123.98, 122.83, 41.22, 31.53, 28.01, 26.77, 22.58, 14.05. HRMS-ESI, calcd. for $C_{42}H_{38}N_2O_4S_4$ [M+]: 762.1709; found: 762.1713.

1.2 NDI-T2-NDI:

Bithiophene (20.0 mg, 120 μmol, 1.0 eq), NDI-Br (124 mg, 241 μmol, 2.0 eq), K_2CO_3 (49.8 mg, 361 μmol, 3.0 eq), and pivalic acid (12.3 mg, 120 μmol, 1.0 eq) were weighed into a dry vial containing a stirring bar. Then 2 mL degassed mesitylene was added under a nitrogen atmosphere and the whole mixture was stirred for 5 min to fully dissolve the monomers. Then Pd_2dba_3 (3.3 mg, 3.61 μmol, 0.03 eq) was added under nitrogen at room temperature. The vial was sealed and placed into a preheated oil bath and stirred for 48 h at 80 °C. After cooling to room temperature,

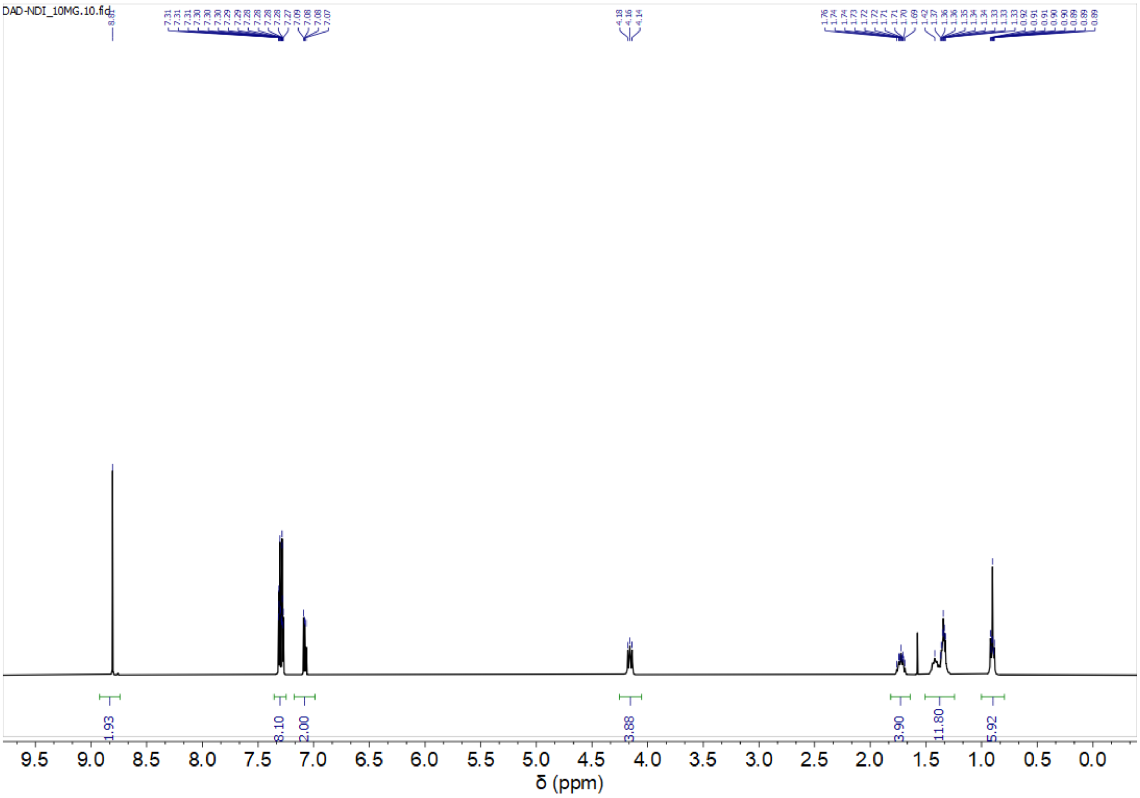

Figure S1.2: ^{1}H NMR spectrum of T2-NDI-T2 in $CDCl_3$.

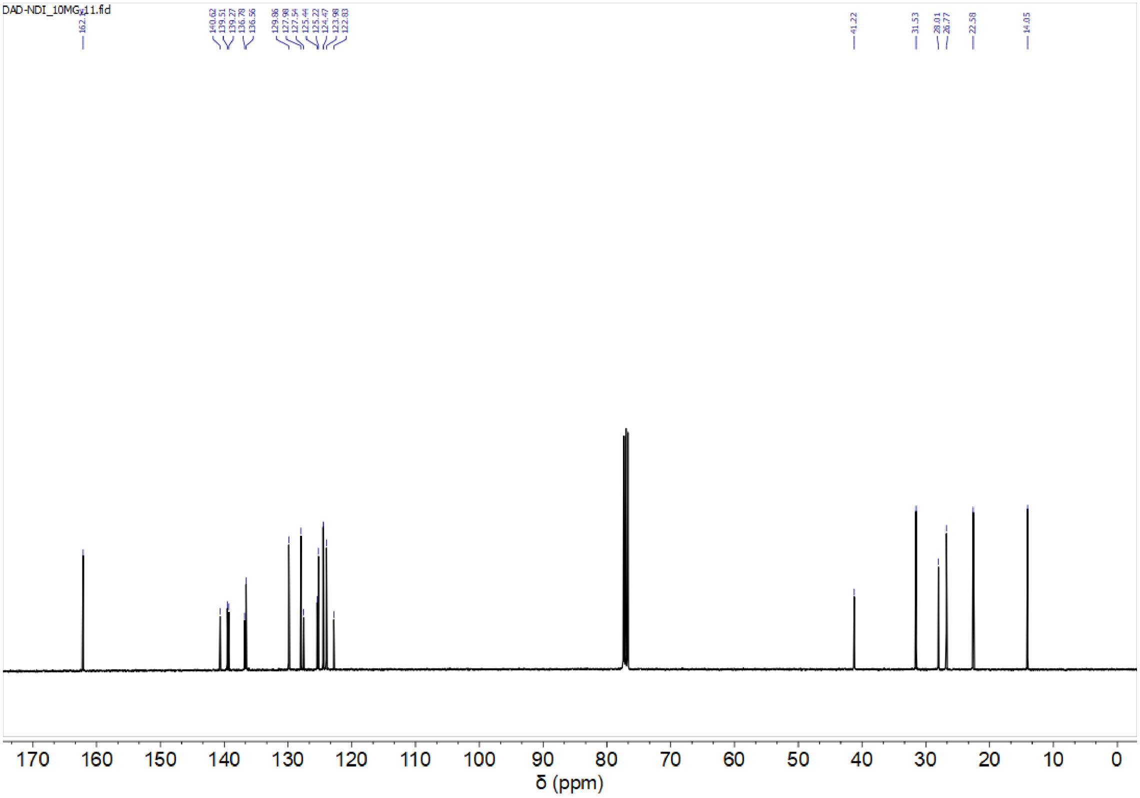

Figure S1.3: ^{13}C^{1}H NMR spectrum of T2-NDI-T2 in $CDCl_3$.

the reaction mixture was directly subjected to column chromatography to yield the desired product (61 mg, 49%). NDI-T2-NDI: ^{1}H NMR (400 MHz, CD_2Cl_2) δ 8.82

(d, J = 7.6 Hz, 2H), 8.77 (d, J = 7.6 Hz, 2H), 8.76 (s, 2H), 7.41 (d, J = 3.8 Hz, 2H), 7.33 (d, J = 3.8 Hz, 2H), 4.21 (appt, J = 7.6 Hz, 4H), 4.16 (appt, J = 7.6 Hz, 4H), 1.83–1.68 (m, 8H), 1.48–1.35 (m, 24H), 0.95–0.86 (m, 12H). ^{13}C NMR (101 MHz, CDCl3) δ 162.78, 162.51, 162.45, 162.07, 140.22, 139.89, 139.74, 136.06, 131.47, 130.62, 129.77, 128.02, 126.83, 126.57, 126.23, 125.35, 124.67, 123.26, 41.25, 41.03, 31.53, 31.50, 28.08, 28.03, 26.80, 26.72, 22.60, 22.54, 14.06, 14.05. HRMS-ESI, calcd. for $C_{60}H_{62}N_4O_8S_2$ [M+]: 1030.4004; found: 1030.4020.

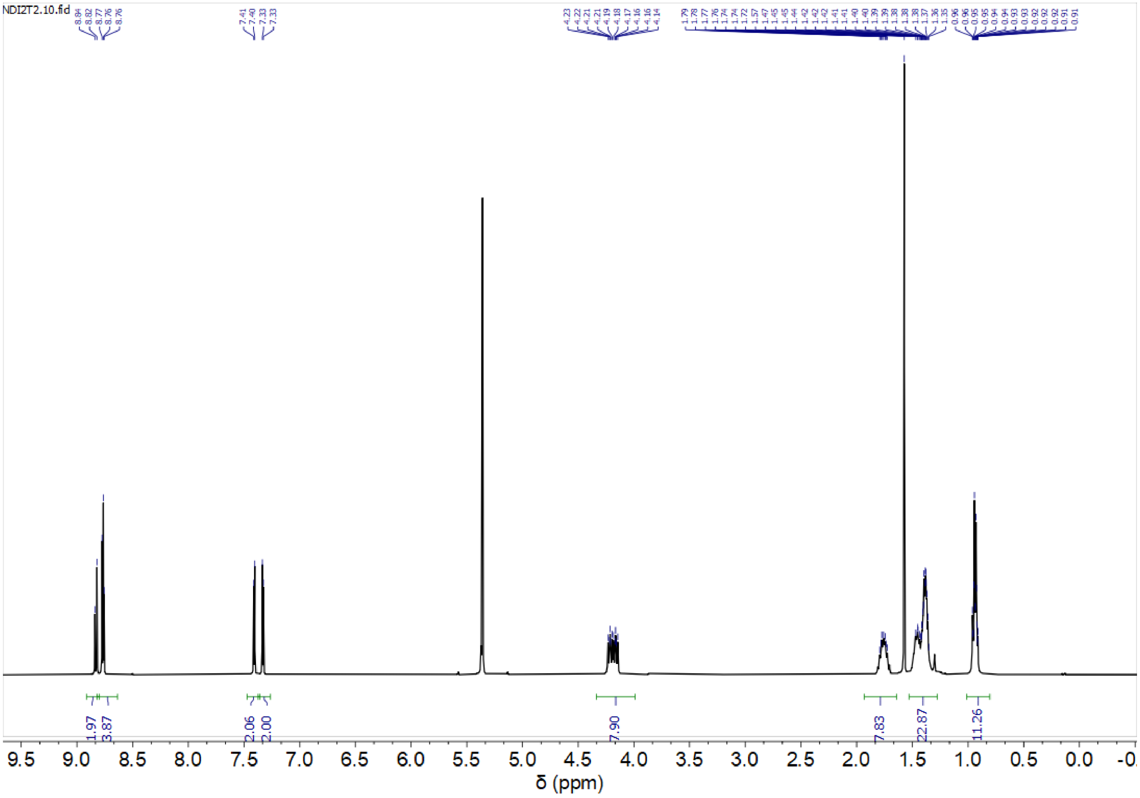

Figure S1.4: ^{1}H NMR spectrum of NDI-T2-NDI in CD_2Cl_2.

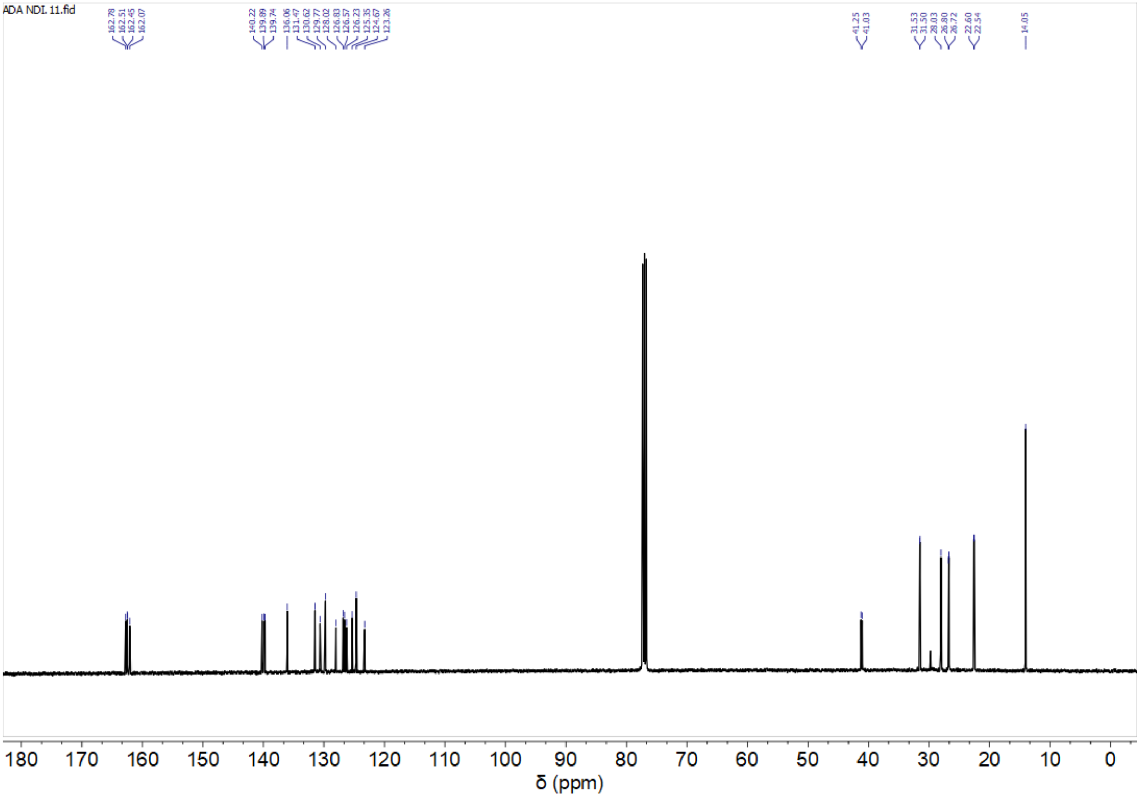

Figure S1.5: ^{13}C NMR spectrum of NDI-T2-NDI in $CDCl_3$.

2 UV-Vis Spectroscopy Details:

The samples for UV-vis spectra were prepared in dry ortho-dichlorobenzene (oDCB) in ambient conditions. The spectra were recorded in transmission mode using a Cary 5000 UV-vis-NIR spectrophotometer in a quartz cuvette with path length of 1 cm. For the molar extinction coefficient experiment, the stock solution of NDI-2T-NDI and 2T-NDI-2T was prepared at 0.1 mM concentration in oDCB and different aliquots of stock were added into oDCB to vary the concentration as follows:

Volume of Stock Solution (μL)	Volume of oDCB (μL)	Final Concentration (μM)
100	2900	3.33
200	2800	6.67
300	2700	10
400	2600	13.3
500	2500	16.7

3 Steady-State and Time-Resolved Photoluminescence:

3.1 Steady-State Luminescence Measurement Details:

Photoluminescence spectra were collected using a custom-built Princeton Instruments spectrometer. A liquid nitrogen-cooled, front-illuminated Si CCD (PyLoN) was used for collecting visible-NIR spectra (425–900 nm) and a 1D liquid-nitrogen cooled InGaAs array (PyLoN-IR) was used for SWIR measurements (850–1550 nm). Vis-NIR spectra were intensity calibrated using an IntelliCal USB-LSVN (9000-410) calibration lamp. SWIR spectra were calibrated using a SWIR quartz tungsten halogen lamp from Princeton Instruments. Dual monochromators (HRS 500) were used to achieve pseudo-monochromatic excitation from an Energetiq EQ99x laser driven light source, with typical FWHM bandwidths *ca.* 16 nm using a 1200 g mm^{-1}, 750 nm blaze grating. A single monochromator was used for detection (Princeton HRS-300) with 1200 g mm^{-1} (500 nm blaze) and 150 g mm^{-1} (800 nm blaze) gratings used for measuring vis-NIR and SWIR spectra, respectively. Typical exposures were 0.5–1 s with 0.25–1 mm detection slit widths. PL spectra for the model compounds and N2200 were collected using 550 nm and 700 nm excitation light respectively.

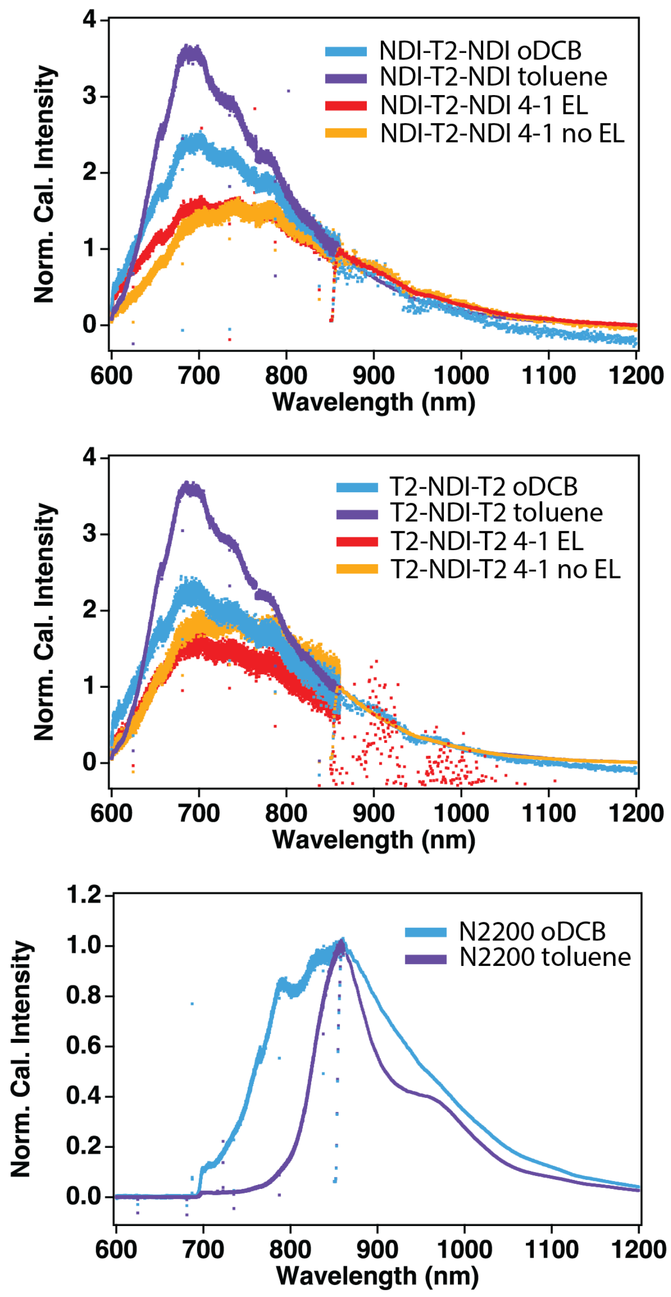

Figure S3.1: Steady-state photoluminescence spectra of model compounds and N2200 in various solvent environments and across both visible-NIR and SWIR detectors, in order to capture entire emission tail out to *ca.* 1200 nm. Spectra are normalized to 1 at the cross-over from the visible-NIR detector to the SWIR detector.

3.2 Strickler-Berg Analysis:

Further details of the Strickler-Berg analysis can be found in the literature.[4] Briefly, the reduced extinction coefficient spectrum was produced by dividing the extinction coefficient at each energy value by the energy in cm^{-1}. The photoluminescence spectrum was bandwidth-corrected by multiplying the intensity at each wavelength by the square of the wavelength and plotting vs. energy. The photoluminescence vs. energy plot was reduced by dividing by the cube of the energy in cm^{-1}. The intrinsic radiative rate constant k_r was then calculated from the reduced extinction coefficient and reduced PL plots as follows:

$$k_r = 2.88x10^{-9}\frac{n_f^3}{n_a} < \nu_f^{-3} >_{avg}^{-1} \int \frac{\epsilon(\nu)}{\nu}d\nu$$

$$< \nu_f^{-3} >_{avg}^{-1} = \frac{\int F(\nu)d\nu}{\int \frac{F(\nu)d\nu}{\nu^3}}$$

where n_f is the refractive index of the medium in which the PL spectrum was taken, n_a that of the medium in which the absorption spectrum was taken, $\int \frac{\epsilon(\nu)}{\nu}d(\nu)$ was taken over the CT band only, and the integrals over the PL spectrum ($\int F(\nu)d\nu$ and $\int \frac{F(\nu)}{\nu^3}d\nu$) were taken over the entire range.

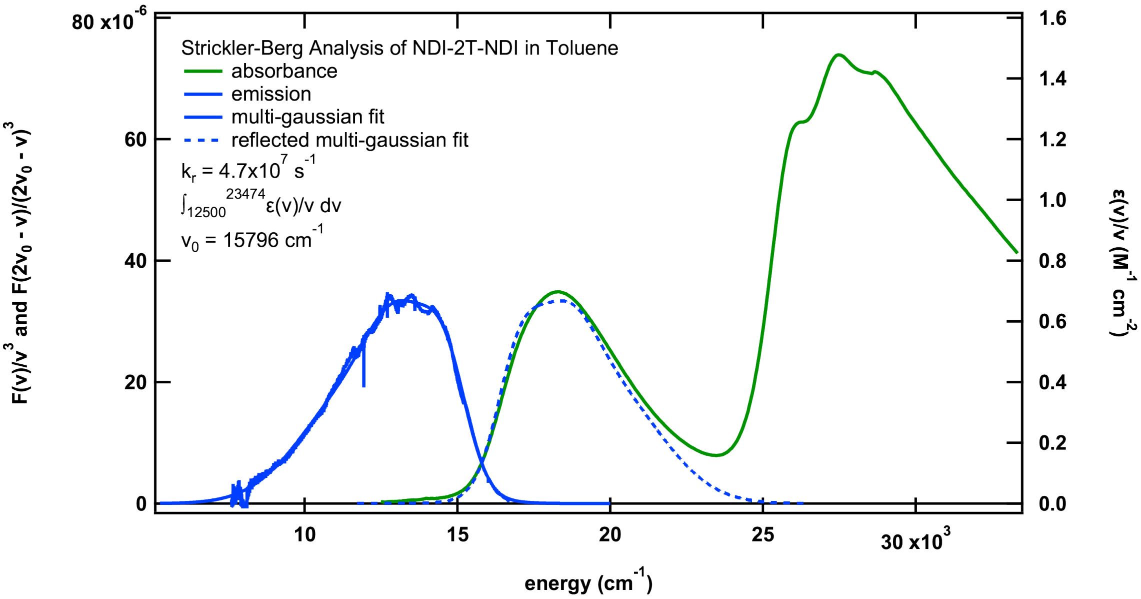

Figure S3.2: Strickler-Berg analysis of NDI-2T-NDI dissolved in toluene.

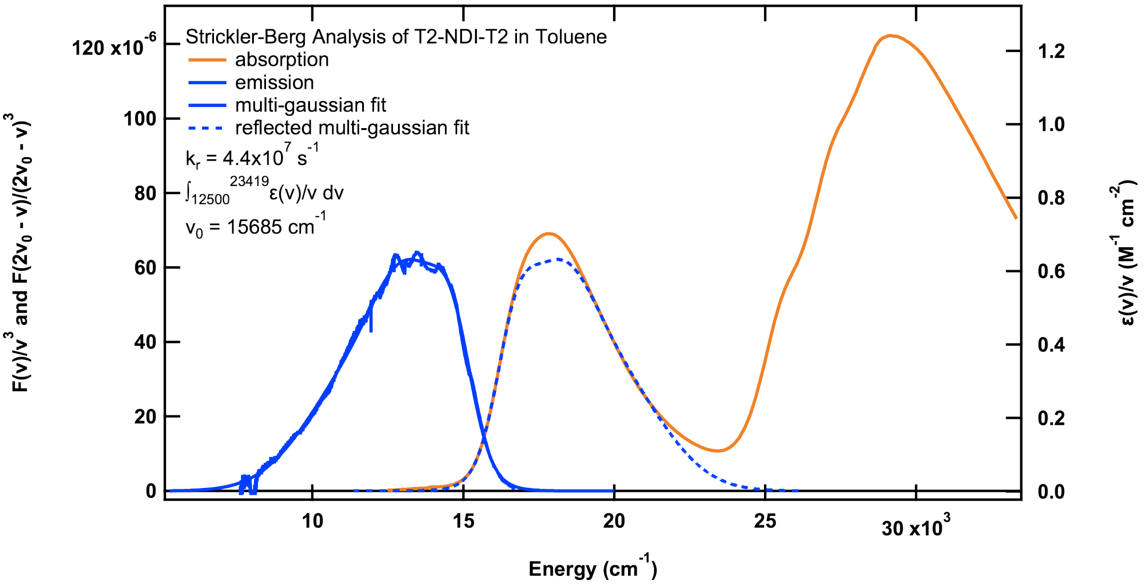

Figure S3.3: Strickler-Berg analysis of T2-NDI-2T dissolved in toluene.

3.3 TRPL Data:

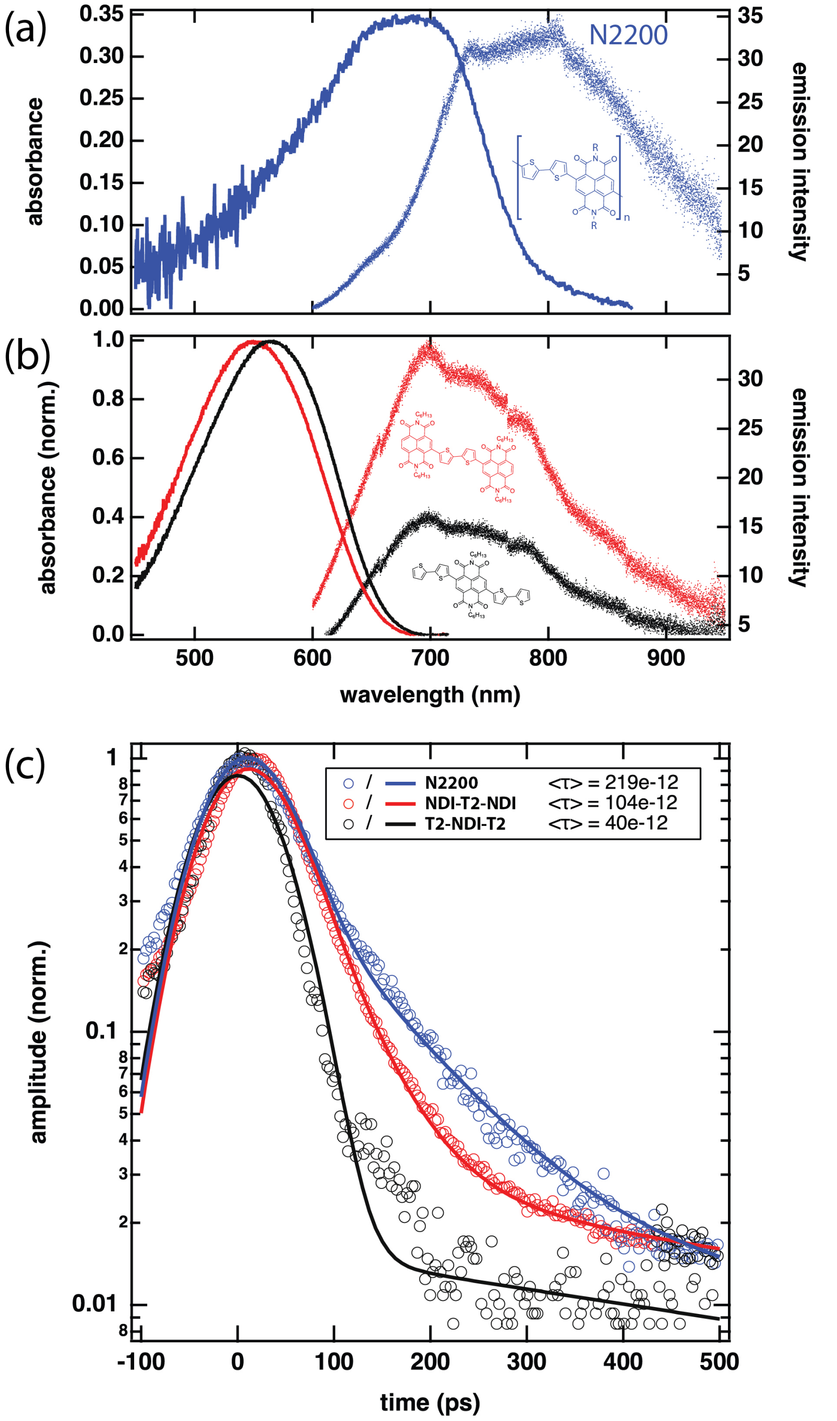

Figure S3.4: (a) absorbance and photoluminescence spectra of N2200 in oDCB. (b) absorbance and photoluminescence spectra of NDI-2T-NDI (red) and T2-NDI-T2 (blue) in oDCB. (c) Time-resolved photoluminescence dynamics of N2200 (blue), NDI-2T-NDI (red), T2-NDI-T2 (black) integrated across their respective emission bands. The time constants given in the inset are the amplitude weighted-average two exponential components in the fit (solid lines)

4 Thin-Film Absorbance:

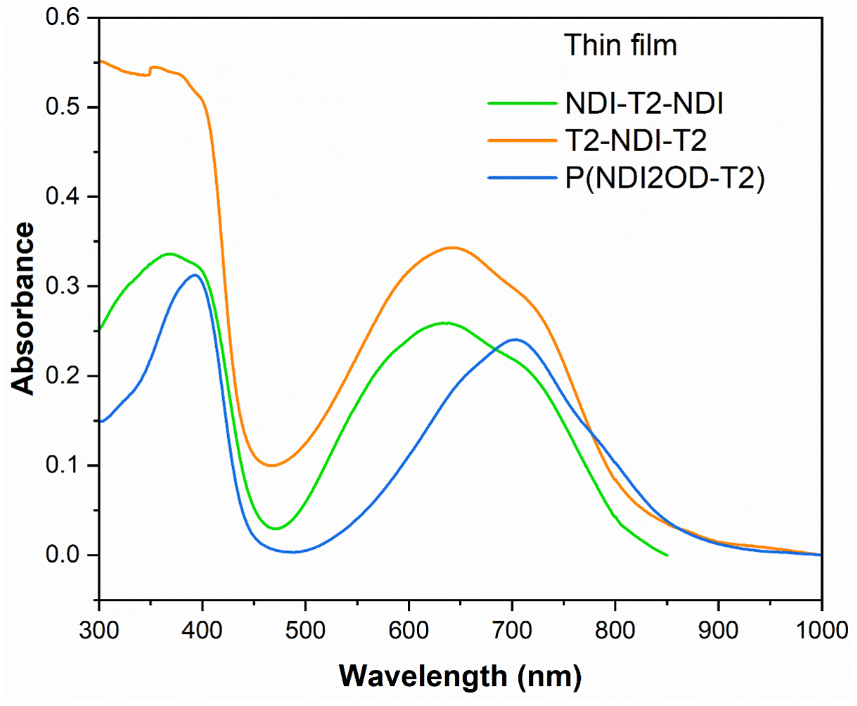

Figure S4.1: Thin film absorption of model compounds and polymer on a quartz substrates recorded in transmission mode using a Cary 7000 spectrophotometer.

5 Cyclic Voltammetry:

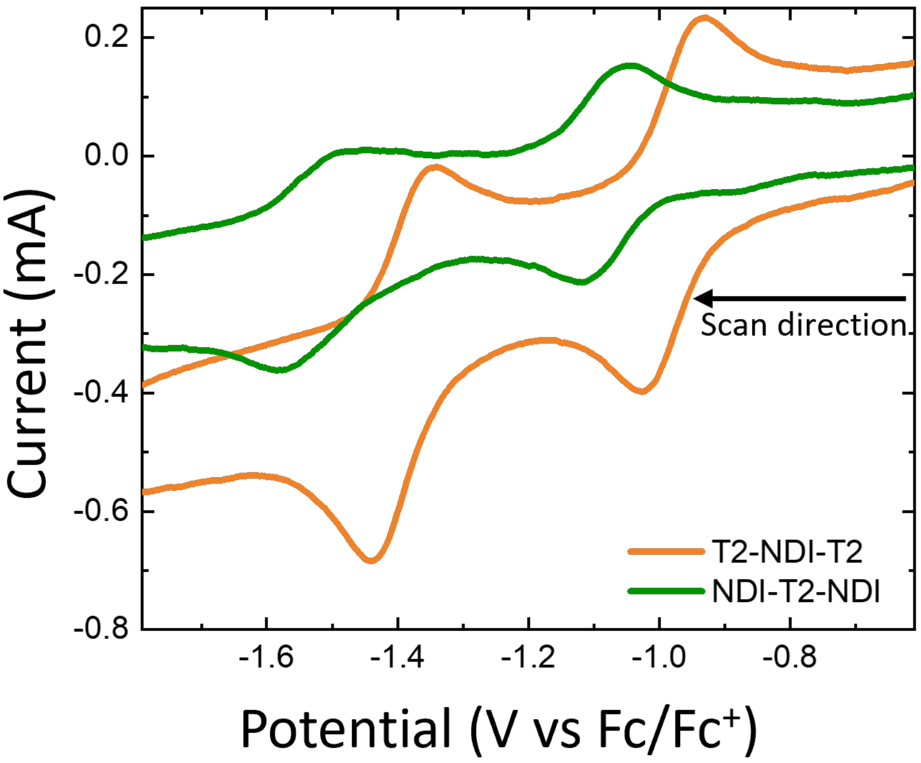

Figure S5.1: Cyclic voltammagrams (IUPAC convention) of T2-NDI-T2 (orange) and NDI-T2-NDI (green) referenced to Fc/Fc$^+$ $E_{1/2}$ in a 4:1 mixture of oDCB:MeCN with 0.1 M $NBu_4{}^+PF_6^-$ supporting electrolyte. Scans began at the open circuit voltage and scanned negative at 20 mV/s.

6 Supplemental Transient Absorption Data:

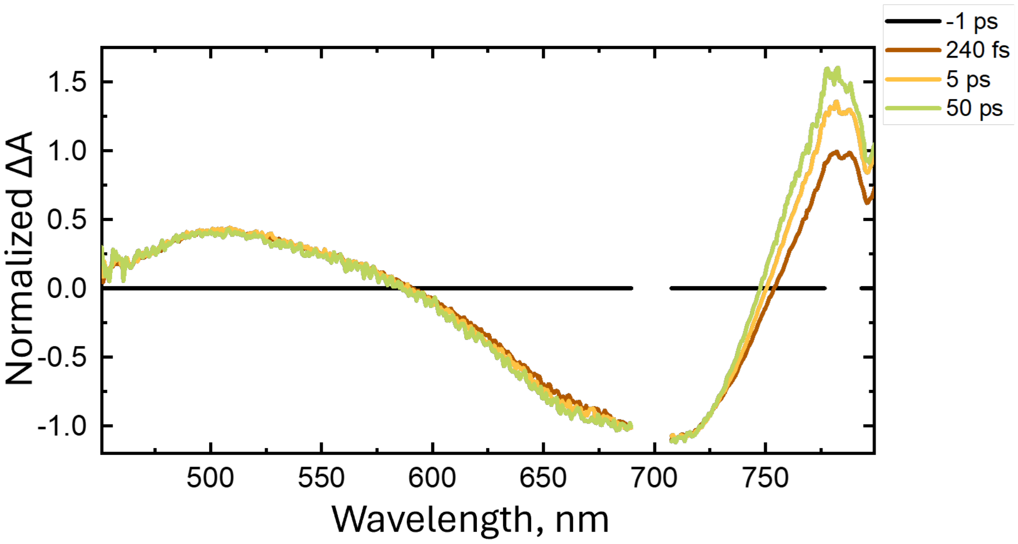

Figure S6.1: Transient absorption spectra of N2200 in oDCB photoexcited at 700 nm. The spectra are normalized to the ground-state bleach at 715 nm to show the pronounced growth of the 760 nm feature assigned to the N2200 polaron.

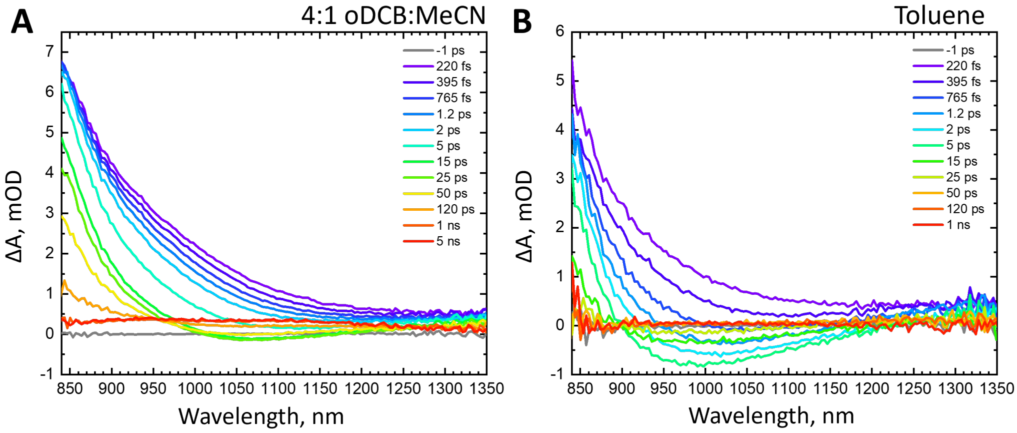

Figure S6.2: Transient absorption spectra of T2-NDI-T2 dissolved in (a) 4:1 oDCB:MeCN and (b) toluene photoexcited at 525 nm (180 nJ/pulse) and probed in the NIR. Representative times are shown in the legend.

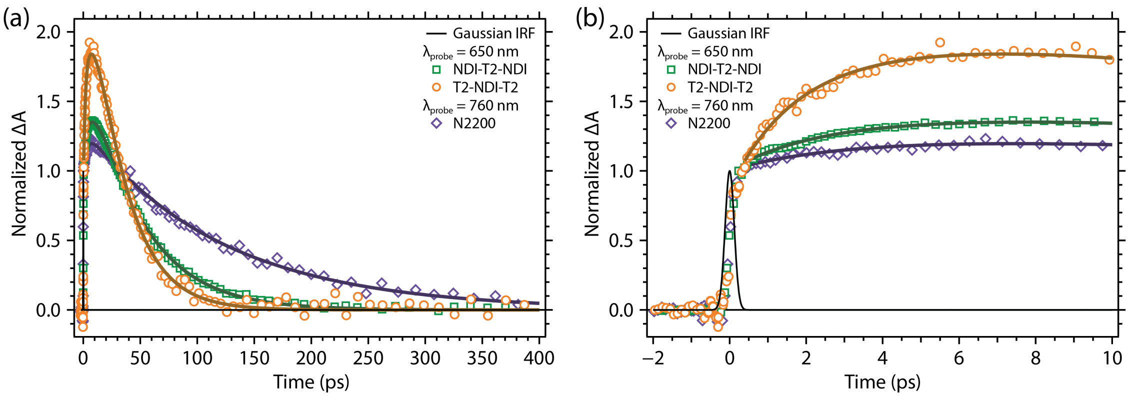

Figure S6.3: Linear-scaled transient absorption spectra of all samples dissolved in oDCB showing short (a) and long (b) timescales

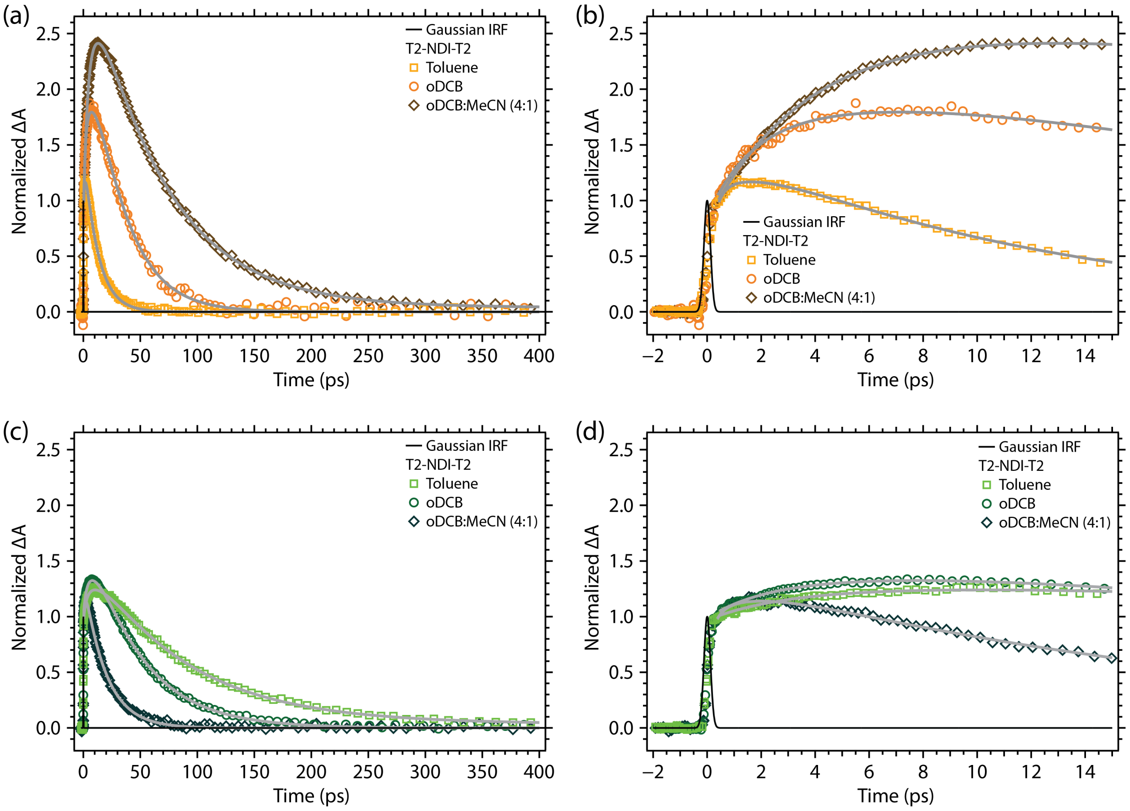

Figure S6.4: Linear-scaled transient absorption spectra of T2-NDI-T2 (a, b) and NDI-T2-NDI (c, d) in toluene, oDCB, and a 4:1 oDCB:MeCN mixture.

Table S6.1: Kinetic fit parameters for the data in Figure 4(b)

$\lambda_{probe} = 650$ nm	NDI-T2-NDI, oDCB:MeCN	NDI-T2-NDI, oDCB	NDI-T2-NDI, toluene
A_1 (rise)	0.16	0.08	0.1
τ_1, ps (rise)	0.28 (±0.2)	1.95 (±0.3)	2.7 (±0.3)
A_2 (rise)	0.17	0.25	0.2
τ_2, ps (rise)	1.6 (±0.3)	9.8 (±0.8)	13.5 (±1.8)
A_3 (decay)	0.67	0.67	0.7
τ_3, ps (decay)	19.3 (±0.2)	45 (±0.6)	85 (±1.5)

Table S6.2: Kinetic fit parameters for the data in Figure 4(a)

$\lambda_{probe} = 650$ nm	T2-NDI-T2, oDCB:MeCN	T2-NDI-T2, oDCB	T2-NDI-T2, toluene
A_1 (rise)	0.24	0.13	0.26
τ_1, ps (rise)	3.4 (±0.17)	1.62 (±0.3)	0.27 (±0.1)
A_2 (rise)	0.20	0.30	0.17
τ_2, ps (rise)	11.3 (±1)	9.7 (±1.6)	1.6 (±0.3)
A_3 (decay)	0.56	0.57	0.57
τ_3, ps (decay)	64 (±0.5)	27 (±1.3)	12 (±0.2)

7 Supplemental Calculations:

Table S7.1: Excitation energies and oscillator strengths of the lowest excited states in NDI-T2, as calculated at the LC-ωhPBE/6-31G(d,p) level of theory with the consideration of o-dichlorobenzene as an implicit solvent.

State	Energy (eV)	Oscillator strength
T_1	2.06	0
T_2	2.06	0
S_1	2.64	0.51
S_2	3.66	0.38

Table S7.2: Excitation energies and oscillator strengths of the lowest excited states in T2-NDI-T2, as calculated at the LC-ωhPBE/6-31G(d,p) level of theory with the consideration of o-dichlorobenzene as an implicit solvent.

	Energy (eV)	Oscillator strength
T_1	1.96	0
T_2	2.33	0
S_1	2.51	0.72
S_2	2.81	0
S_3	3.70	0.35

Table S7.3: Excitation energies and oscillator strengths of the lowest excited states in NDI-T2-NDI, as calculated at the LC-ωhPBE/6-31G(d,p) level of theory with the consideration of o-dichlorobenzene as an implicit solvent.

	Energy (eV)	Oscillator strength
T_1	2.10	0
T_2	2.24	0
S_1	2.65	0.62
S_2	2.83	0.04

Table S7.4: Frequencies (ω), relaxation energies (L), and Huang-Rhys (S) factors related to the $S_0 \rightarrow S_1$ and $S_1 \rightarrow S_0$ transitions in T2-NDI. The calculations are performed at the TD-DFT/LC-ωhPBE/6-31G(d,p) level of theory with the consideration of o-dichlorobenzene as an implicit solvent.

$S_0 \rightarrow S_1$			$S_1 \rightarrow S_0$		
ω (cm^{-1})	L (cm^{-1})	S	ω (cm^{-1})	L (cm^{-1})	S
13	4.65	0.359	17	2.09	0.120
21	10.12	0.477	27	46.37	1.733
23	31.74	1.390	29	41.63	1.415
46	5.75	0.126	54	0.61	0.011
54	18.62	0.342	57	44.48	0.785
60	88.62	1.467	68	5.91	0.087

71	8.27	0.117	74	9.22	0.125
77	6.22	0.081	88	102.49	1.168
89	33.71	0.381	96	0.41	0.004
99	83.86	0.844	100	128.44	1.285
113	46.07	0.406	117	19.23	0.164
121	5.99	0.050	124	41.73	0.336
135	5.21	0.039	140	26.02	0.185
145	168.83	1.161	150	186.14	1.239
175	112.90	0.644	185	168.05	0.907
189	0.00	0.000	196	27.04	0.138
208	1.75	0.008	203	2.13	0.010
225	35.89	0.160	211	27.57	0.131
259	0.04	0.000	264	0.04	0.000
264	10.81	0.041	277	28.19	0.102
298	70.45	0.237	299	52.29	0.175
310	5.29	0.017	307	63.12	0.206
317	0.02	0.000	315	8.00	0.025
318	8.78	0.028	323	1.97	0.006
345	3.48	0.010	344	11.98	0.035
365	6.34	0.017	358	14.24	0.040
373	8.31	0.022	372	2.08	0.006
390	13.98	0.036	388	47.86	0.124
399	53.69	0.135	402	42.45	0.106
408	8.67	0.021	410	2.84	0.007
418	0.11	0.000	419	0.25	0.001
450	0.01	0.000	435	0.12	0.000
456	0.07	0.000	452	1.18	0.003
467	0.79	0.002	470	0.21	0.000
486	2.58	0.005	475	0.87	0.002
511	2.70	0.005	505	22.52	0.045
529	0.94	0.002	523	1.52	0.003
550	4.40	0.008	550	10.50	0.019
571	0.20	0.000	562	0.00	0.000
576	8.62	0.015	572	0.80	0.001
578	38.82	0.067	576	55.58	0.096
599	0.00	0.000	592	3.31	0.006
601	0.08	0.000	612	0.25	0.000
628	0.22	0.000	623	1.97	0.003
629	1.37	0.002	627	0.03	0.000
660	0.00	0.000	638	4.98	0.008
680	5.23	0.008	677	11.71	0.017
710	20.60	0.029	712	8.23	0.012
715	0.49	0.001	714	28.11	0.039
722	11.14	0.015	715	3.32	0.005
744	6.40	0.009	740	0.54	0.001
747	1.09	0.001	741	0.06	0.000

757	10.52	0.014	744	3.77	0.005
762	6.61	0.009	746	8.67	0.012
766	0.34	0.000	755	8.57	0.011
772	0.47	0.001	756	2.29	0.003
774	0.04	0.000	774	0.48	0.001
783	4.70	0.006	777	0.44	0.001
822	5.42	0.007	803	0.61	0.001
841	14.31	0.017	844	19.02	0.023
849	5.74	0.007	859	2.10	0.002
862	5.71	0.007	859	6.42	0.007
867	13.44	0.015	871	0.07	0.000
904	0.01	0.000	876	17.63	0.020
911	0.01	0.000	907	13.53	0.015
915	19.92	0.022	924	0.55	0.001
929	0.03	0.000	943	2.44	0.003
958	0.06	0.000	946	0.01	0.000
963	2.17	0.002	955	1.85	0.002
973	0.77	0.001	972	1.06	0.001
1015	0.00	0.000	997	0.00	0.000
1029	0.15	0.000	1032	0.12	0.000
1059	2.24	0.002	1057	1.20	0.001
1073	0.62	0.001	1068	0.08	0.000
1080	19.47	0.018	1092	25.53	0.023
1088	9.59	0.009	1105	3.39	0.003
1107	0.55	0.001	1110	0.03	0.000
1116	3.82	0.003	1125	6.12	0.005
1149	0.04	0.000	1149	0.02	0.000
1150	0.00	0.000	1150	0.00	0.000
1165	54.25	0.047	1166	39.60	0.034
1190	11.93	0.010	1192	15.38	0.013
1208	0.24	0.000	1202	13.71	0.011
1231	11.53	0.009	1227	15.55	0.013
1242	10.88	0.009	1246	31.40	0.025
1254	0.94	0.001	1253	0.55	0.000
1261	0.06	0.000	1257	2.44	0.002
1276	34.95	0.027	1277	68.37	0.054
1296	1.40	0.001	1311	5.99	0.005
1336	3.63	0.003	1320	0.04	0.000
1342	8.56	0.006	1329	2.40	0.002
1347	20.03	0.015	1346	0.26	0.000
1375	16.64	0.012	1373	4.84	0.004
1387	16.25	0.012	1387	22.19	0.016
1411	0.01	0.000	1415	0.10	0.000
1418	0.76	0.001	1423	0.17	0.000
1432	0.83	0.001	1430	27.11	0.019
1436	9.69	0.007	1438	8.87	0.006

1458	0.00	0.000	1454	30.60	0.021
1476	45.48	0.031	1468	17.63	0.012
1482	103.68	0.070	1478	192.43	0.130
1487	0.02	0.000	1483	2.91	0.002
1488	13.82	0.009	1487	0.01	0.000
1499	0.03	0.000	1488	0.00	0.000
1504	0.30	0.000	1495	0.32	0.000
1507	5.37	0.004	1501	5.67	0.004
1515	21.02	0.014	1506	1.76	0.001
1543	429.69	0.278	1514	7.52	0.005
1573	32.15	0.020	1537	310.47	0.202
1597	181.76	0.114	1559	61.44	0.039
1639	9.64	0.006	1568	0.91	0.001
1647	50.30	0.031	1576	0.53	0.000
1680	107.73	0.064	1633	217.94	0.133
1700	12.55	0.007	1647	28.27	0.017
1766	36.20	0.021	1664	328.73	0.198
1769	12.32	0.007	1731	1.68	0.001
1808	51.35	0.028	1750	57.90	0.033
1815	148.83	0.082	1774	91.06	0.051
3096	0.02	0.000	3091	0.05	0.000
3096	0.13	0.000	3091	0.11	0.000
3177	0.00	0.000	3168	0.00	0.000
3178	0.00	0.000	3170	0.00	0.000
3228	0.00	0.000	3223	0.00	0.000
3228	0.00	0.000	3223	0.00	0.000
3234	0.00	0.000	3226	0.00	0.000
3237	0.03	0.000	3234	0.00	0.000
3239	0.01	0.000	3246	0.00	0.000
3240	0.01	0.000	3248	0.01	0.000
3249	0.00	0.000	3254	0.05	0.000
3254	0.21	0.000	3267	0.21	0.000
3259	1.78	0.001	3289	0.00	0.000
3287	0.00	0.000	3291	0.13	0.000
Total relaxation energy (cm^{-1})	2558		3054		

Table S7.5: Atomic cartesian coordinates of the NDI-T2 molecule at the optimized S_1-state geometry and the nonadiabatic coupling (NAC) constants between the S_1 and S_0 states, as obtained at the LC-ωHPBE/6-31G** level of theory with the consideration of o-dichlorobenzene as an implicit solvent.

Atoms	Coordinates (Å)			NAC ($bohr^{-1}$)		
	X	Y	Z	X	Y	Z
C	-0.5612	-1.3217	0.0692	0.1466	0.1658	0.1071
C	-0.0208	-0.0309	0.0771	-0.2744	-0.1375	0.0244
C	-0.8928	1.0975	-0.0018	-0.0575	-0.2287	-0.1110

C	-2.2940	0.8946	-0.0031	0.0861	-0.0452	-0.0110
C	-2.8233	-0.4279	0.0327	0.1814	-0.1025	0.0096
C	-1.9335	-1.5341	0.0507	-0.1626	0.0692	-0.0273
C	-3.1883	1.9838	-0.0594	-0.1409	-0.0134	-0.0188
C	-4.5697	1.7678	-0.0512	0.1356	0.1268	0.0020
C	-5.0785	0.4785	-0.0052	0.0497	-0.1927	0.0025
C	-4.2130	-0.6198	0.0301	-0.1411	0.0374	0.0010
C	-4.7690	-1.9829	0.0606	-0.0077	-0.0231	0.0095
N	-3.8491	-3.0322	0.0856	-0.0130	0.0164	0.0037
C	-2.4574	-2.8950	0.0815	-0.0032	-0.0069	-0.0262
C	-0.3753	2.4329	-0.1889	0.1086	0.2125	-0.0724
N	-1.2891	3.4869	-0.2002	0.0155	-0.0114	0.0922
C	-2.6752	3.3557	-0.1335	-0.0006	0.1046	0.0145
O	-5.9765	-2.1993	0.0638	-0.0012	0.0094	-0.0037
O	-1.7471	-3.8978	0.1041	-0.0061	0.0190	0.0034
O	0.8324	2.6934	-0.3378	-0.1613	-0.0661	-0.0238
O	-3.4080	4.3422	-0.1540	0.0328	-0.0674	-0.0136
H	0.0875	-2.1908	0.1386	0.0013	0.0075	0.0007
H	-5.2262	2.6315	-0.0873	0.0027	0.0006	0.0050
C	7.6578	-0.9351	-0.1182	0.0589	-0.0648	-0.0511
S	6.5878	0.1031	0.7148	-0.0343	0.0070	0.0100
C	5.2055	-0.6207	-0.0639	0.0638	-0.0757	-0.0531
C	5.6104	-1.6292	-0.9340	-0.0506	0.0334	0.0189
C	7.0042	-1.8050	-0.9606	0.0722	0.0383	0.0455
C	3.8848	-0.1799	0.2109	-0.0227	0.1156	0.0901
C	3.4670	0.7972	1.1243	0.1108	-0.1178	-0.1090
C	2.0958	0.9743	1.1453	-0.1531	-0.0924	0.0043
C	1.4069	0.1273	0.2437	0.3894	0.1283	0.0605
S	2.5180	-0.8667	-0.6326	-0.0388	0.0764	0.0268
H	4.9148	-2.2170	-1.5242	-0.0039	0.0013	0.0001
H	7.5158	-2.5390	-1.5720	0.0042	-0.0023	-0.0021
H	4.1577	1.3392	1.7609	0.0050	0.0053	-0.0037
H	1.5734	1.6453	1.8139	0.0006	0.0045	0.0011
C	-0.7267	4.8235	-0.3412	0.0060	0.0108	0.0058
H	-0.0066	5.0095	0.4587	-0.0040	-0.0005	-0.0005
H	-0.2114	4.9161	-1.3008	-0.0019	-0.0034	-0.0008
H	-1.5497	5.5333	-0.2857	0.0027	-0.0003	-0.0004
H	-6.1483	0.2943	-0.0029	-0.0007	-0.0016	-0.0008
H	8.7241	-0.8522	0.0522	-0.0006	0.0017	-0.0015
C	-4.3556	-4.3972	0.1174	-0.0029	-0.0019	0.0009
H	-4.0057	-4.9463	-0.7602	0.0002	0.0013	0.0001
H	-3.9926	-4.9088	1.0121	0.0000	0.0012	-0.0002
H	-5.4426	-4.3475	0.1240	0.0004	-0.0005	0.0001

Table S7.6: Frequencies (ω), relaxation energies (L) and Huang-Rhys (S) factors related to the $S_1 \rightarrow T_1$ and $S_1 \rightarrow T_1$ transitions in T2-NDI. The calculations are performed at the LC-ωhPBE/6-31G(d,p) level of theory with the consideration of o-dichlorobenzene as an implicit solvent.

	$S_1 \rightarrow T_1$			$T_1 \rightarrow S_1$	
ω (cm^{-1})	S	L(cm^{-1})	ω (cm^{-1})	S	L (cm^{-1})
20	10.092	200.23	23	11.625	262.44
26	5.029	128.69	30	0.813	24.76
31	0.441	13.58	32	4.337	137.08
57	0.002	0.12	52	0.004	0.22
58	0.437	25.52	57	0.537	30.37
72	0.660	47.83	67	0.808	53.90
80	0.032	2.52	73	0.502	36.69
90	0.007	0.64	85	0.006	0.48
97	0.000	0.01	97	0.104	10.03
102	0.048	4.92	101	0.275	27.71
121	0.005	0.60	116	0.008	0.91
125	0.005	0.57	122	0.008	1.00
141	0.000	0.02	146	0.003	0.50
151	0.011	1.65	149	0.010	1.45
186	0.000	0.06	190	0.084	16.01
197	0.047	9.18	192	0.032	6.11
204	0.028	5.64	202	0.118	23.72
212	0.053	11.14	216	0.024	5.18
264	0.020	5.31	265	0.014	3.83
278	0.021	5.81	268	0.000	0.03
300	0.000	0.07	295	0.014	4.17
307	0.012	3.76	300	0.005	1.51
315	0.007	2.34	309	0.006	1.70
324	0.013	4.18	324	0.020	6.41
345	0.002	0.61	342	0.035	12.09
358	0.001	0.26	355	0.060	21.27
373	0.003	1.13	371	0.001	0.41
388	0.000	0.12	387	0.005	1.84
403	0.000	0.03	404	0.002	0.68
410	0.002	0.63	408	0.002	0.83
420	0.000	0.03	416	0.000	0.12
436	0.002	0.70	435	0.006	2.67
452	0.014	6.41	452	0.005	2.36
470	0.001	0.63	463	0.001	0.24
476	0.003	1.41	475	0.001	0.36
506	0.003	1.69	509	0.005	2.77
524	0.005	2.80	512	0.001	0.53
551	0.000	0.23	539	0.005	2.58
562	0.001	0.29	555	0.000	0.13
572	0.001	0.31	572	0.000	0.08

578	0.003	1.87	579	0.002	1.15
594	0.006	3.60	582	0.008	4.74
613	0.014	8.59	603	0.015	9.23
624	0.012	7.78	617	0.000	0.30
629	0.000	0.16	622	0.004	2.73
639	0.002	1.17	635	0.000	0.02
680	0.002	1.11	673	0.012	8.15
712	0.001	0.68	703	0.004	2.92
718	0.003	2.31	712	0.002	1.14
719	0.000	0.17	717	0.000	0.02
740	0.000	0.08	725	0.000	0.02
742	0.000	0.16	741	0.000	0.02
744	0.000	0.03	745	0.000	0.15
747	0.000	0.05	752	0.000	0.01
756	0.001	0.87	755	0.001	0.39
758	0.001	0.62	758	0.002	1.43
774	0.000	0.00	776	0.000	0.03
778	0.001	0.74	778	0.003	2.43
804	0.002	1.25	783	0.008	6.26
846	0.000	0.35	838	0.001	1.04
859	0.000	0.01	839	0.000	0.14
860	0.003	2.90	858	0.001	0.54
872	0.000	0.00	871	0.001	1.14
878	0.001	0.96	874	0.000	0.01
910	0.002	1.57	906	0.003	2.34
924	0.004	3.44	920	0.000	0.05
942	0.005	4.28	938	0.000	0.00
946	0.000	0.01	947	0.005	4.37
957	0.001	1.29	949	0.000	0.09
974	0.000	0.13	971	0.000	0.01
997	0.000	0.00	1001	0.000	0.01
1033	0.002	1.57	1031	0.000	0.29
1058	0.001	1.36	1061	0.000	0.02
1069	0.001	0.58	1072	0.001	1.50
1093	0.001	0.60	1086	0.000	0.30
1106	0.001	1.19	1106	0.000	0.00
1111	0.000	0.00	1109	0.000	0.26
1127	0.007	7.91	1121	0.008	9.15
1149	0.000	0.01	1150	0.000	0.02
1150	0.000	0.00	1150	0.000	0.00
1167	0.000	0.14	1168	0.001	0.85
1192	0.004	4.23	1182	0.002	1.89
1204	0.001	1.64	1196	0.002	2.28
1228	0.001	1.27	1219	0.003	4.06
1247	0.000	0.30	1224	0.005	6.17
1254	0.000	0.11	1251	0.000	0.24

1258	0.000	0.17	1255	0.000	0.15
1277	0.000	0.04	1268	0.001	1.18
1312	0.001	1.36	1305	0.006	7.33
1321	0.001	1.36	1326	0.000	0.05
1330	0.003	4.16	1331	0.000	0.35
1348	0.006	8.15	1343	0.000	0.06
1373	0.003	3.59	1351	0.005	6.25
1389	0.008	10.65	1377	0.002	2.88
1415	0.000	0.04	1393	0.006	8.52
1422	0.002	2.25	1413	0.000	0.01
1431	0.002	2.64	1423	0.001	1.12
1438	0.010	13.76	1435	0.001	1.71
1457	0.004	6.12	1444	0.006	8.78
1470	0.002	3.01	1456	0.005	7.36
1480	0.002	2.52	1475	0.002	2.93
1484	0.003	4.56	1479	0.000	0.70
1487	0.000	0.04	1487	0.000	0.20
1489	0.001	1.05	1487	0.000	0.00
1495	0.002	2.38	1491	0.000	0.62
1501	0.001	1.31	1499	0.000	0.40
1508	0.000	0.04	1506	0.000	0.71
1515	0.000	0.40	1513	0.001	0.78
1544	0.053	81.12	1548	0.049	76.16
1560	0.002	3.20	1551	0.013	20.39
1570	0.020	32.13	1561	0.005	7.36
1578	0.003	4.21	1612	0.019	30.30
1635	0.000	0.09	1641	0.013	21.87
1648	0.001	0.95	1659	0.006	10.69
1667	0.001	1.10	1691	0.004	6.61
1731	0.000	0.81	1749	0.001	0.87
1751	0.001	2.16	1760	0.001	2.48
1775	0.000	0.73	1790	0.000	0.33
3091	0.000	0.01	3094	0.000	0.04
3091	0.000	0.04	3094	0.000	0.03
3168	0.000	0.00	3173	0.000	0.00
3169	0.000	0.00	3174	0.000	0.00
3223	0.000	0.00	3219	0.000	0.00
3223	0.000	0.00	3226	0.000	0.00
3224	0.000	0.01	3227	0.000	0.00
3233	0.000	0.00	3238	0.000	0.00
3245	0.000	0.03	3244	0.000	0.03
3245	0.000	0.02	3246	0.000	0.00
3251	0.000	0.01	3250	0.000	0.03
3266	0.000	0.00	3263	0.000	0.00
3288	0.000	0.04	3289	0.000	0.03
3289	0.000	0.10	3304	0.000	0.00

Total relaxation energy (cm^{-1})	755		Total relaxation energy (cm^{-1})	1012	

Table S7.7: Spin-orbit coupling constants (in cm-1) between the lowest singlet and triplet states in T2-NDI computed at the LC-ωhPBE/6-31G(d,p) level of theory with the consideration of o-dichlorobenzene as an implicit solvent. The calculations were performed at the ground-state geometry.

	T_1	T_2	T_3
S_0	4.4	5.6	5.7
S_1	3.0	2.4	2.0
S_2	7.3	2.9	2.3

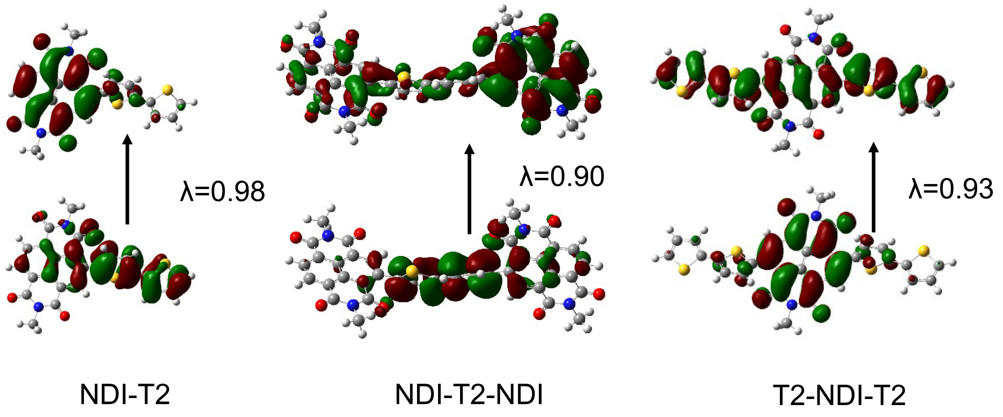

Figure S7.1: Natural transition orbitals (NTOs) as calculated at the LC-ωhPBE/6-31G(d,p) level of theory for the electronic transition between the ground state and lowest singlet excited state (S_1) in T2-NDI, NDI-T2-NDI, and T2-NDI-T2, with the consideration of o-dichlorobenzene as an implicit solvent.

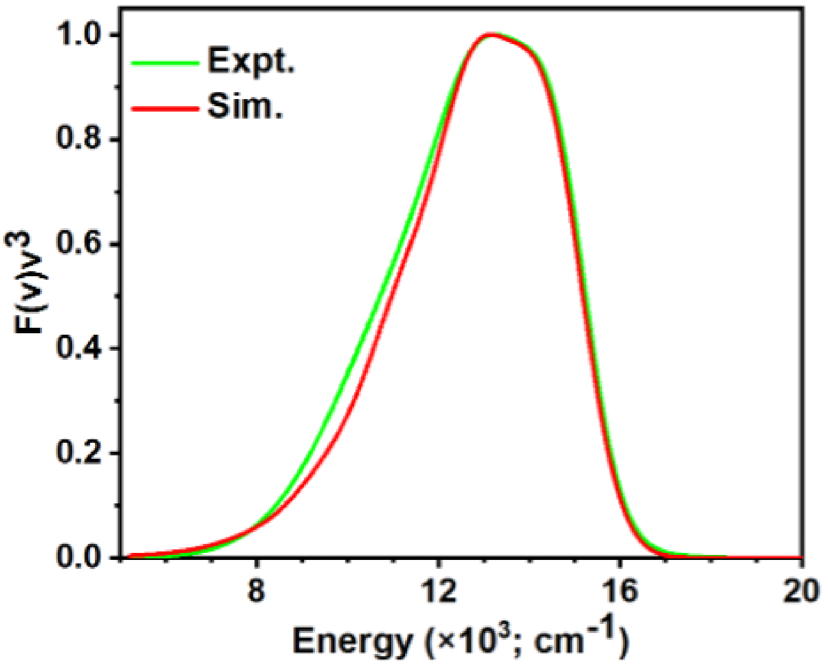

Figure S7.2: Experimental and simulated line-shapes of the reduced emission of NDI-T2-NDI. The experimental band was measured in toluene. The theoretical curve was obtained by fitting the experimental line-shape in the framework of the Marcus-Levich-Jortner model. The following best fitting microscopic parameters were obtained (see methodology section): λ_c=0.20 eV, Sqm=1.027, ω_{qm}=1600 cm^{-1}, E_{CT} = 1.98 eV, and T=300 K.

References

(1) Matsidik, R.; Komber, H.; Brinkmann, M.; Schellhammer, K. S.; Ortmann, F.; Sommer, M. *J. Am. Chem. Soc.* **2023**, *145*, 8430–8444.

(2) Polander, L. E.; Tiwari, S. P.; Pandey, L.; Seifried, B. M.; Zhang, Q.; Barlow, S.; Risko, C.; Brédas, J. L.; Kippelen, B.; Marder, S. R. *Chem. Mater.* **2011**, *23*, 3408–3410.

(3) Polander, L. E.; Romanov, A. S.; Barlow, S.; Hwang, D. K.; Kippelen, B.; Timofeeva, T. V.; Marder, S. R. *Org. Lett.* **2012**, *14*, 918–921.

(4) Mitchell, W. J.; Ferguson, A. J.; Köse, M. E.; Rupert, B. L.; Ginley, D. S.; Rumbles, G.; Shaheen, S. E.; Kopidakis, N. *Chem. Mater.* **2008**, *21*, 287–297.
